# Competitive cocrystallization and its application in the separation of flavonoids

**DOI:** 10.1107/S2052252520015997

**Published:** 2021-01-21

**Authors:** Yanming Xia, Yuanfeng Wei, Hui Chen, Shuai Qian, Jianjun Zhang, Yuan Gao

**Affiliations:** a School of Traditional Chinese Pharmacy, China Pharmaceutical University, Nanjing 211198, People’s Republic of China; b School of Pharmacy, China Pharmaceutical University, Nanjing 211198, People’s Republic of China

**Keywords:** cocrystals, flavonoids, separation methods, intermolecular interactions

## Abstract

Recently, cocrystallization has been widely employed to tailor physicochemical properties of drugs in the pharmaceutical field. In this study, cocrystallization was applied to separate natural compounds with similar structures. Three flavonoids [baicalein (BAI), quercetin (QUE) and myricetin (MYR)] were used as model compounds. The coformer caffeine (CAF) could form cocrystals with all three flavonoids, namely BAI–CAF (cocrystal 1), QUE–CAF (cocrystal 2) and MYR–CAF (cocrystal 3). After adding CAF to methanol solution containing MYR and QUE (or QUE and BAI), cocrystal 3 (or cocrystal 2) preferentially formed rather than cocrystal 2 (or cocrystal 1), indicating that flavonoid separation could be achieved by competitive cocrystallization. After co-mixing the slurry of two flavonoids with CAF followed by centrifugation, the resolution ratio that could be achieved was 70–80% with purity >90%. Among the three cocrystals, cocrystal 3 showed the lowest formation constant with a negative Gibbs free energy of nucleation and the highest energy gap. Hirshfeld surface analysis and density of states analysis found that cocrystal 3 had the highest strong interaction contribution and the closest electronic density, respectively, followed by cocrystal 2 and cocrystal 1, suggesting CAF could competitively form a cocrystal with MYR much more easily than QUE and BAI. Cocrystallization is a promising approach for green and effective separation of natural products with similar chemical structures.

## Abstract 2

Competitive cocrystallization provides a green chemical method for flavonoid separation with fewer steps and lower energy consumption than the traditional organic solvent extraction method.

## 1. Introduction   

The first cocrystal, quinhydrone, was discovered by Friedrich Wohler in 1844, but its complete structure and intermolecular interactions were unknown until 1958 (Karagianni *et al.*, 2018 ▸). Pharmaceutical cocrystals are crystalline materials composed of two or more different molecules, typically an active pharma­ceutical ingredient and cocrystal formers, in the same crystal structure (Aitipamula *et al.*, 2012 ▸; Grothe *et al.*, 2016 ▸). Cocrystallization has been widely applied to tailor pharmaceutical related properties of drug candidates, such as solubility (Good & Rodríguez-Hornedo, 2009 ▸; Kalepu & Nekkanti, 2015 ▸), dissolution (Babu & Nangia, 2011 ▸; Shekhawat & Pokharkar, 2017 ▸), bioavailability (Huang *et al.*, 2014 ▸; Jung *et al.*, 2010 ▸), stability (Trask *et al.*, 2006 ▸) as well as compressibility (Krishna *et al.*, 2015 ▸). Entresto, approved by the US Food and Drug Administration (FDA) in 2015 for the treatment of chronic heart failure, is the first commercial cocrystal with the combination of two BCS II drugs, sacubitril and valsartan (Bolla & Nangia, 2016 ▸; Karagianni *et al.*, 2018 ▸). Such a drug–drug cocrystal not only increases the solubility of both drugs, but also creates a dual-drug synergistic formulation. Now, drug cocrystals are increasingly popular in both academia (Bolla & Nangia, 2016 ▸) and industry [*e.g.* Epilim (Petruševski *et al.*, 2008 ▸), Tramadol–Celecoxib (Almansa *et al.*, 2017 ▸; Videla *et al.*, 2017 ▸) and TAK-020 (Esfandiari *et al.*, 2018 ▸)].

Recently, another important application of cocrystals is separating chiral compounds, which are resolved by two cocrystallizing components either forming a cocrystal pair of diastereomers or behaving with enantiomeric specificity (Springuel & Leyssens, 2012 ▸). For instance, Harmsen & Leyssens (2018 ▸) used levetiracetam to resolve racemic ibuprofen via the formation of an enantiospecific cocrystal. He *et al.* (2018 ▸) separated the racemic ofloxacin by forming diastereomeric cocrystal pairs with tartaric acid derivatives.

**Figure 1:**
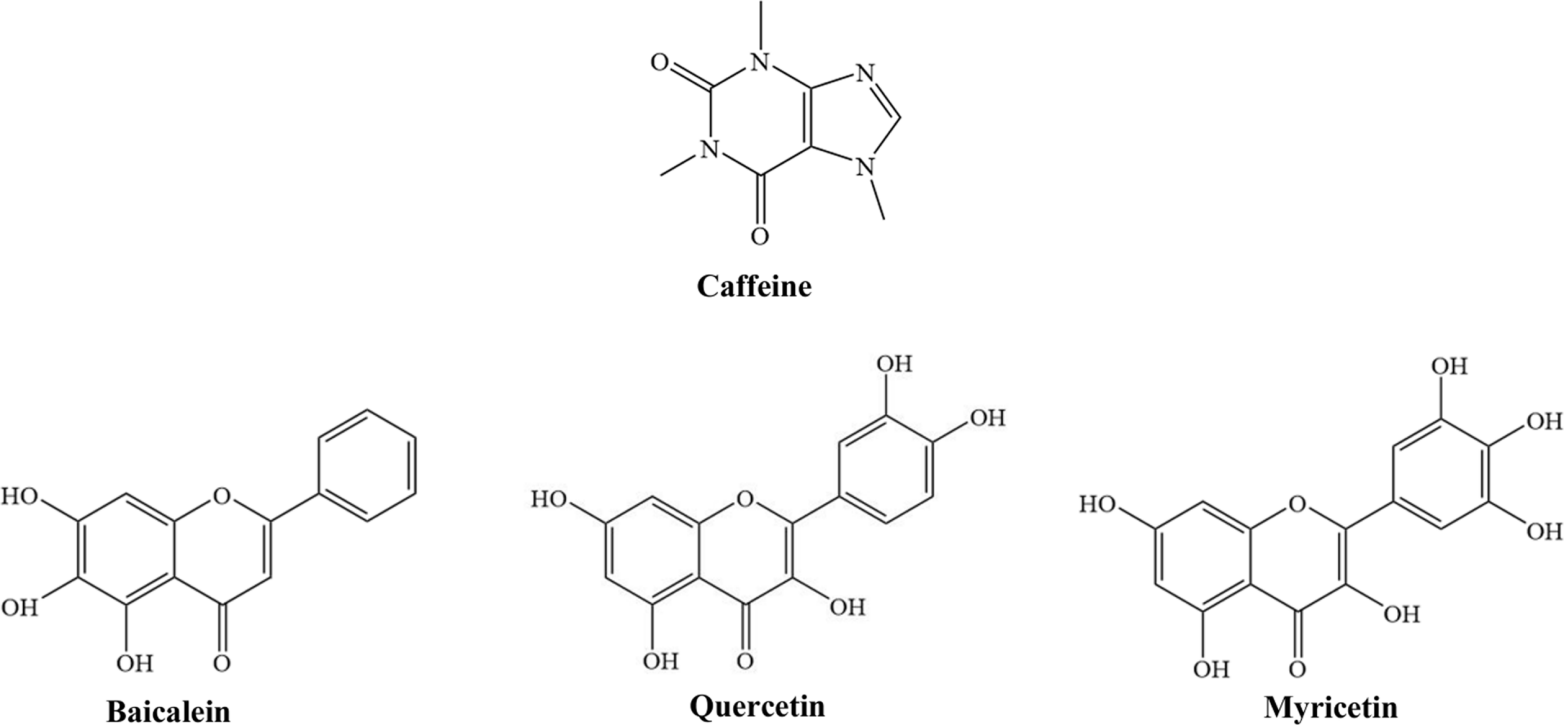
Molecular structures of caffeine and the three flavonoids.

During the exploration of natural products, the separation and purification of structurally similar compounds have become the most important issues (Ren *et al.*, 2013 ▸; Janas *et al.*, 2016 ▸; Zhang *et al.*, 2010 ▸). Solvent extraction (Pyo *et al.*, 2009 ▸), column liquid chromatography and capillary electrophoresis (Liu *et al.*, 2014 ▸) are common approaches for chemical separation. However, the disadvantages of these techniques have aroused much concern, such as the risk of irreversible sorption, low recoveries and environmentally unfriendly consumption, with a great amount of organic solvent used (Wu, *et al.*, 2018*a* ▸). For instance, baicalein (BAI), quercetin (QUE) and myricetin (MYR) are flavonoids with only a difference in the number or sites of hydroxyl groups (Fig. 1 ▸). Currently, conventional methods, such as column chromatography, preparative/semipreparative reversed-phase liquid chromatography, macroporous resin and traditional liquid–liquid extraction, are used for the isolation and purification of flavonoids (Ding *et al.*, 2013 ▸). However, these methods require multiple chromatographic steps that are not only cumbersome, but also time-consuming and organic solvent-consuming. In the current study, we attempted to explore the potential of cocrystallization for the separation of these chemically similar natural products, and the internal separation mechanism was also investigated by molecular modeling.

## 2. Experimental   

### 2.1. Drugs and reagents   

Baicalein (99.0% purity), quercetin (99.0% purity) and myricetin (98.0% purity) were obtained from Nanjing Zelang Medical Technology Company, Ltd (Nanjing, China). Caffeine (CAF) (99.3% purity) was provided by Jiangxi Kangfeng Biological Technology Corporation (Jiangxi, China). Methanol (HPLC grade) was purchased from E. Merck (Darmstadt, Germany). Phosphoric acid (HPLC grade) was purchased from Aladdin Bio-Chem Technology Co., Ltd (Shanghai, China).

### 2.2. Preparation of samples   

#### 2.2.1. Preparation of cocrystals   

The baicalein–caffeine (2:1) cocrystal (BAI–CAF, cocrystal 1) and quercetin–caffeine (1:1) cocrystal (QUE–CAF, cocrystal 2) were prepared by a previously reported slurry method (Zhu *et al.*, 2017 ▸; Smith *et al.*, 2011 ▸). In brief, 1.45 g of CAF and 4.03 g BAI (or 2.54 g QUE) were separately weighed and added into a glass vial containing 50 ml methanol, and stirred at room temperature for 12 h. All bulk samples were filtered and vacuum dried at 25°C for 24 h, the resulting solids were sieved through a 200 mesh (∼75 µm) and stored in a vacuum desiccator at 4°C for further study.

A single crystal of the myricetin–caffeine (1:1) cocrystal (MYR–CAF, cocrystal 3) was successfully obtained by the slow evaporation method. In brief, 10.04 mg of MYR and 6.09 mg of CAF were dissolved in methanol (5 ml) and filtered through a 0.22 µm filter, the filtrate was then evaporated at room temperature in a fume hood. Cocrystal 3 was harvested and a single crystal of appropriate size was selected for structural analysis. The powder crystal of cocrystal 3 was prepared by the same method and used for cocrystals 1 and 2 as described above.

#### 2.2.2. Competitive cocrystallization and separation   

In order to investigate competitive cocrystallization of flavonoids with a coformer, CAF was added in the slurry containing BAI and QUE (or QUE and MYR) for reaction. The solids obtained were characterized to identify which one or both flavonoids cocrystallized with the coformer. Briefly, CAF (1 mmol) was added in the 10 ml methanol slurry containing 2 mmol BAI and 1 mmol QUE or 1 mmol QUE and 1 mmol MYR, after stirring for 12 h at room temperature. In order to analyze the composition, the solids obtained after filtration were vacuum dried and stored in a vacuum desiccator at 4°C for further characterization.

In order to separate the uncrystallized flavonoids, a series of volumes (*i.e.* 10, 20, 30, 40, 50, 60, 100 and 200 ml) of methanol were added to the product after stirring for 12 h. After vortexing for 5 min, the solid–liquid mixture was separated by centrifugation, and the contents of flavonoids in the precipitate collected were analyzed by a validated HPLC.

BAI, QUE, MYR and CAF were separated from each other and baseline-separated simultaneously with retention times of 10.1, 5.6, 7.9 and 3.9 min, respectively, by a Welch Xtimate C_18_ column (6.0 mm × 150 mm, 5 µm) on an LC-2010AHT Shimadzu HPLC system (Shimadzu Corporation). The mobile phase, consisting of 0.1%(*v*/*v*) phosphoric acid aqueous solution (*A*) and methanol solution (*B*), was run at a flow rate of 1.0 ml min^−1^. The gradient program was set as follows: 3 min 60% *B*, 5 min 70% *B*, 9 min 85% *B* and 15 min 95% *B*. The wavelength of detection was set at 254 nm and the column temperature was set at 30°C. The injection volume was 10 µl. Within the concentration range 0.1–3 m*M* for all analytes, good linearities (*r* ^2^ > 0.999) were achieved. For each analyte, the intra-day and inter-day precision relative standard deviation (RSD) values were below 2.6%, and the accuracy was within the range 97–103%.

### 2.3. Physicochemical characterizations   

#### 2.3.1. Differential scanning calorimetry   

Thermal behaviors of samples were obtained using a differential scanning calorimeter (DSC 204F1 Phoenix, NETZSCH, Germany). About 3–5 mg powder samples were placed in the aluminium pan and heated from 25 to 330°C with a rate of 10°C min^−1^. The data were processed by the NETZSCH *Proteus* software (version 4.2).

#### 2.3.2. Powder X-ray diffraction   

Powder X-ray diffraction (PXRD) analyses were carried out on a Bruker D8 Advance X-ray diffractometer with a Cu *K*α radiation source (λ = 1.5406 Å). The tube voltage and amperage were set at 40 kV and 40 mA, respectively. Samples were placed in an aluminium holder and their PXRD patterns were collected with a step size of 0.02° and a scanning speed of 10° min^−1^ over a 2θ range from 3 to 40°. The data were analyzed using the *Origin* software (version 9.0, OriginLab, Northampton, Massachusetts, USA).

#### 2.3.3. Fourier transform infrared spectroscopy   

Fourier transform infrared (FT-IR) spectra of samples were recorded by a Shimadzu IRAffinity-1S infrared spectrometer. About 2–3 mg samples were mixed with KBr (200 mg) and pressed into a slice by a hydraulic press. A total of 64 scans were performed in the range 4000–400 cm^−1^ (with a spectral resolution of 4 cm^−1^). The data were processed by Nicolet Omnic 8.0 infrared spectroscopy processing software.

#### 2.3.4. Single-crystal X-ray diffraction   

The single-crystal X-ray diffraction (SCXRD) data of the MYR–CAF cocrystal were collected in an Enraf Nonius CAD-4 single-crystal diffractometer equipped with Mo *K*α radiation (λ = 0.71073 Å). The structure was determined by direct methods. Structure refinement was carried out using the full-matrix least-squares methods implemented in the *SHELX* program. All non-hydrogen atoms and anisotropic temperature factors were structurally corrected using the full-matrix least-squares method.

### 2.4. Solubility of flavonoid cocrystals   

In order to calculate the cocrystal formation constant (*K*_c_), the solubility of cocrystals in solution with different concentrations of CAF were investigated (Good & Rodríguez-Hornedo, 2009 ▸, 2010 ▸). In brief, excess of three flavonoid cocrystals solid powders were separately added into glass-stoppered bottles containing CAF methanol solution of various concentrations (0.05, 0.1, 0.2, 0.4, 0.5, 0.8 and 1.0 mg ml^−1^) and stirred at 25°C for 48 h. The supernatant was filtered and analyzed by HPLC.

### 2.5. Data analysis   

#### 2.5.1. Calculation of separation factors   

The separation efficiency of flavonoids A and B was calculated by the separation factor α, and the flavonoid analog excess value (%ae) (He *et al.*, 2018 ▸). The two parameters can be calculated by the following equations:
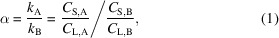


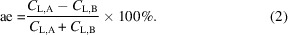
where *C*_S,A_ and *C*_S,B_ represent the concentrations of flavonoids A and B in the solid phase after separation, respectively. *C*_L,A_ and *C*_L,B_ represent the concentrations of flavonoids A and B in the liquid phase after separation, respectively.

#### 2.5.2. Calculation of *K*_c_ for cocrystals   

The theory of the formation constant (*K*_c_) can be used to explain the formation mechanism of a cocrystal (Zhang *et al.*, 2007 ▸). For the molar ratio of a 1:1 cocrystal (such as cocrystal 3), when there is no complex effect between MYR and CAF, the cocrystal 3 solution equilibria in methanol solution could be described as follows:
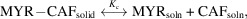


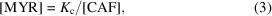
where [CAF] and [MYR] are the molar concentrations of CAF and MYR, respectively. Linear regression of [MYR] versus 1/[CAF] is a straight line passing through the origin, and the slope value is *K*_c_.

When there is a 1:1 complex formed between MYR and CAF, the equilibria between MYR and CAF in methanol could be described as follows (Ma *et al.*, 2014 ▸; Nehm *et al.*, 2006 ▸):
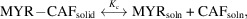


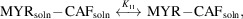


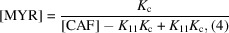
where the *K*_11_ is the 1:1 complexation constant between MYR and CAF.

For the molar ratio of a 2:1 cocrystal (such as cocrystal 1), when there is no complex effect between BAI and CAF, the cocrystal 1 solution equilibria in methanol is similar to the 1:1 cocrystal; when forming a 1:1 and/or 2:1 solution complex, three situations will appear as follows (Xu *et al.*, 2011 ▸): for a 1:1 complex only
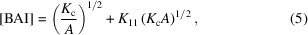
where
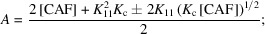
and for a 2:1 complex only
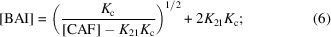
1:1 and 2:1 complexes:
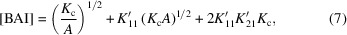
where
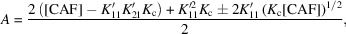
where [BAI] and [CAF] are the molar concentrations of BAI and CAF in equilibrium with the cocrystal, *K*_11_ and *K*_21_ are the complexation constants of cocrystal 1 in the 1:1 and 2:1 solution complex model, and *K*′_11_ and *K*′_21_ are the complexation constants of cocrystal 1 in 1:1 and 2:1 solution complex models.

#### 2.5.3. Calculation of Gibbs free energy   

According to the cocrystal reaction formula, the Gibbs free energy (Δ*G*°) can be calculated as follows:
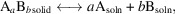


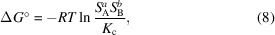
where *S*_A_ and *S*_B_ represent the solubility of pure A and B, respectively.

### 2.6. Molecular simulations   

#### 2.6.1. *Blends* module analysis   

In order to investigate the solvent effects and thermodynamic mixing variables between the various molecular models, the compound pairs were calculated in the *Blends* module (*Materials Studio* 8.0). All cocrystals and solvent molecular models were mechanics-optimized by the COMPASS force field, improved Flory–Huggins theory and molecular simulation techniques were used to calculate the interaction parameters of specified molecules in different combinations, and the force field setting was the same as the geometry optimization (Pajula *et al.*, 2010 ▸).

#### 2.6.2. Hirshfeld surface analysis   

The intermolecular interactions of cocrystals were investigated using Hirshfeld surfaces and 2D fingerprint plots. The function *d*_norm_ is a ratio encompassing the distances of any surface point to the nearest interior (*d*_i_) and exterior (*d*_e_) atom, as well as the van der Waals radii of the atoms. A plot of *d*_i_ versus *d*_e_ is a 2D fingerprint plot which recognizes the existence of intermolecular interactions of different types (Spackman & Jayatilaka, 2009 ▸). The Hirshfield surfaces of all cocrystals were calculated using the *CrystalExplorer* 3.1 program, the density functional theory (DFT) methodology with the B3LYP density functional and the 6-31G(d,p) basis set were employed.

#### 2.6.3. Electronic structure analysis   

For a more accurate cocrystal electronic structure, density of states (DOS) calculations were performed with the *CASTEP* module (*Materials Studio* 8.0) using the plane-wave pseudopotential method (PWP) within the formalism of DFT (Feng *et al.*, 2017 ▸). The exchange-associative interactions were described by a generalized gradient approximation (GGA) combined with the Perdew–Burke–Ernzerh (PBE) method. The detailed parameters of the cocrystal electronic structure and partial density of states (PDOS) were found in these studies.

### 2.7. Energy calculations   

The energy values of cocrystal molecular structures have been calculated by gradient-corrected DFT using the B3LYP method 6-31+G(d,p) basis set. The atomic coordinates obtained for the crystal structure were used for computing. Frontier molecular orbital analysis was carried out to predict the chemical stability. The *GaussView05* program (Denning­ton *et al.*, 2009 ▸) was used for molecular electrostatic potential mapping analysis. Using default convergence principles, DFT calculations were performed at the ground-state energy level without affecting any constraints on the molecular geometry using the *Gaussian16* program package (Li *et al.*, 2020 ▸).

## 3. Results   

### 3.1. Cocrystal synthesis   

**Figure 2:**
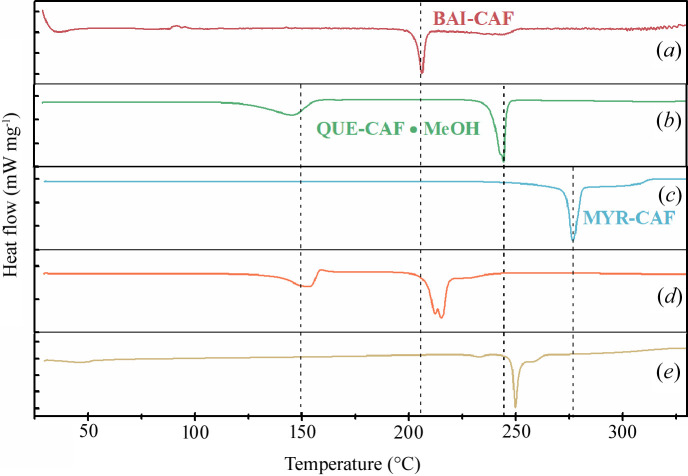
DSC profiles of the cocrystals (*a*) BAI–CAF, (*b*) QUE–CAF·MeOH, (*c*) MYR–CAF) and competitive systems (*d*) BQC, (*e*) QMC.

The differential scanning calorimetry (DSC) data of the slurry products of BAI:CAF (2:1), QUE:CAF (1:1) and MYR:CAF (1:1) are shown in Fig. 2 ▸. The DSC thermogram of BAI:CAF (2:1) showed that there was a heat absorption melting peak at 206.1°C [Fig. 2 ▸(*a*)], which was consistent with that reported in the literature (Zhu *et al.*, 2017 ▸). The QUE:CAF (1:1) slurry product exhibited two endothermal peaks at 146.1 and 244.7°C, which could be attributed to the loss of an MeOH molecule and the melting point, respectively [Fig. 2 ▸(*b*)]. Such thermal behavior was in accordance with that reported in the literature (Smith *et al.*, 2011 ▸). Cocrystal 3 formed by the methanol slurry method only had a single sharp peak at 276.7°C [Fig. 2 ▸(*c*)], which was a new substance different from the physical mixture of MYR and CAF (271.5 and 273.8°C, see Fig. S1 of the supporting information), indicating the formation of the cocrystal.

**Figure 3:**
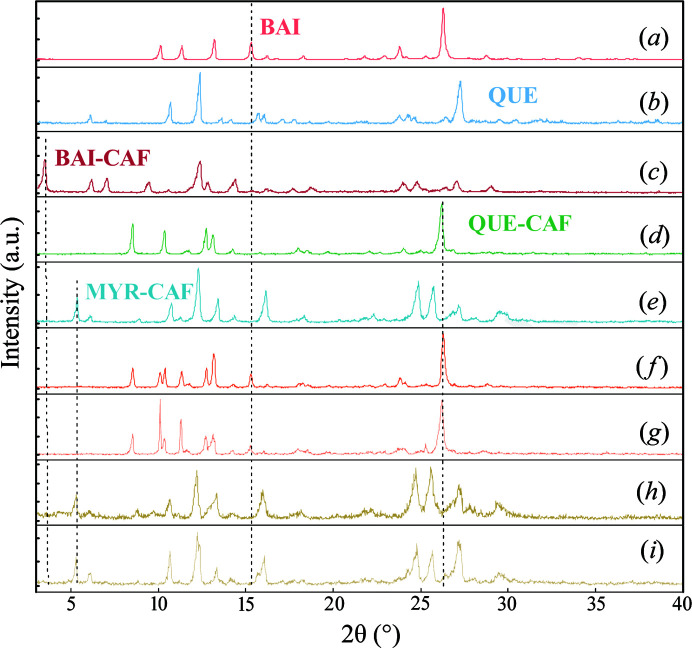
PXRD diffractograms of crystals (*a*) BAI and (*b*) QUE; slurry products of the three cocrystals (*c*) BAI–CAF, (*d*) QUE–CAF, (*e*) MYR–CAF; and competitive systems (*f*) BQC, (*h*) QMC; and physical mixtures of crystal and cocrystal (*g*) BAI:QUE–CAF 1:1, (*i*) QUE:MYR–CAF 1:1.

The PXRD measurement results of the slurry products of the three cocrystals were shown in Fig. 3 ▸. The cocrystal 1 characteristic diffraction peaks appeared at 3.5, 7.0, 9.4, 14.2 and 23.8° 2θ [Fig. 3 ▸(*c*)], cocrystal 2 had characteristic diffraction peaks at 8.5, 10.4, 12.7, 13.1 and 26.3° 2θ [Fig. 3 ▸(*d*)], which were identical to those reported in the literature (Smith *et al.*, 2011 ▸; Zhu *et al.*, 2017 ▸). The diffractogram of cocrystal 3 was distinguishable from the physical mixture of MYR and CAF (Fig. S2), and showed sharp diffraction peaks at 5.2, 10.6, 12.2, 24.7 and 25.6° 2θ [Fig. 3 ▸(*e*)] with its corresponding pattern simulation produced by a single-crystal structure [Figs. S3(*e*) and S3(*f*)], indicating the formation of cocrystal 3 in a 1:1 molar ratio.

**Figure 4:**
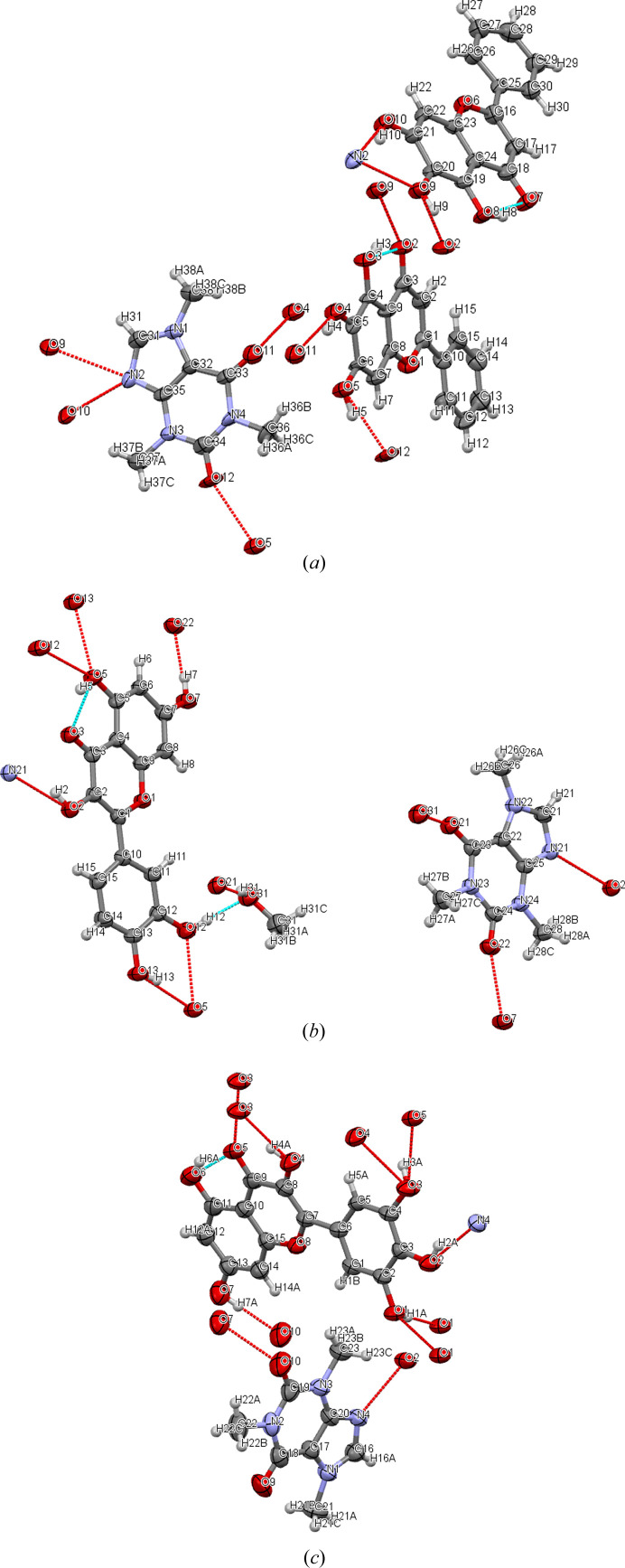
Schematic motif of the cocrystals with hydrogen bonds (*a*) BAI–CAF, (*b*) QUE–CAF·MeOH, (*c*) MYR–CAF. Hydrogen bonds between molecules forming heterodimers are indicated by red lines, and blue dashed lines represent the inter- and intramolecular hydrogen bonds of the flavonoids.

The structures of cocrystals 1 and 2 [Figs. 4 ▸(*a*) and 4(*b*)] were obtained from the literature (Smith *et al.*, 2011 ▸; Zhu *et al.*, 2017 ▸) and crystallographic data (Table 1 ▸) were downloaded from the Cambridge Crystallographic Data Centre (CCDC). Cocrystal 3 was cocrystallized by the slurry method and the single crystal was harvested by slow solvent evaporation. SCXRD data of cocrystal 3 are shown in Table 1 ▸. Cocrystal 3 contains four molecules of MYR and CAF in the unit cell and crystallizes in the space group *P*2_1_/*n*. The CAF molecule adopts an almost planar conformation, with the hydrogen bonds O_3_—H_3A_⋯O_5_ (distance 2.746 Å), O_1_—H_1A_⋯O_1_ (distance 2.943 Å) and O_4_—H_4A_⋯O_3_ (distance 3.004 Å). Two available hydroxyl substituents on the benzopyran ring in the MYR molecule act as hydrogen-bond donors to connect CAF via O_2_—H_2A_⋯N_4_ (distance 2.790 Å) and O_7_—H_7A_⋯O_10_ (distance of 2.689 Å) hydrogen bonds [Fig. 4 ▸(*c*)]. The spatial arrangement of cocrystal 3 and the supramolecular inter­actions between the cocrystal molecules are shown in Fig. S9(*c*).

### 3.2. Cocrystal competitive reaction   

The DSC thermograms of the BAI, QUE and CAF mixture system slurry products (BQC mixture) are shown in Fig. 2 ▸. The BQC [Fig. 2 ▸(*d*)] mixture exhibited a wide endothermic peak at ∼149.1°C, which might be considered as the de-methanol peak, suggesting that cocrystal 2 may exist in this system. In addition, the BQC system exhibited two endotherms with melting points at 206.6 and 215.5°C, whereas the QUE, MYR and CAF mixture system slurry products (QMC) [Fig. 2 ▸(*e*)] exhibited two endotherms with melting points at 249.8 and 271.4°C, respectively, which infers that the two competitive systems were mixtures composed of different substances.

**Figure 5:**
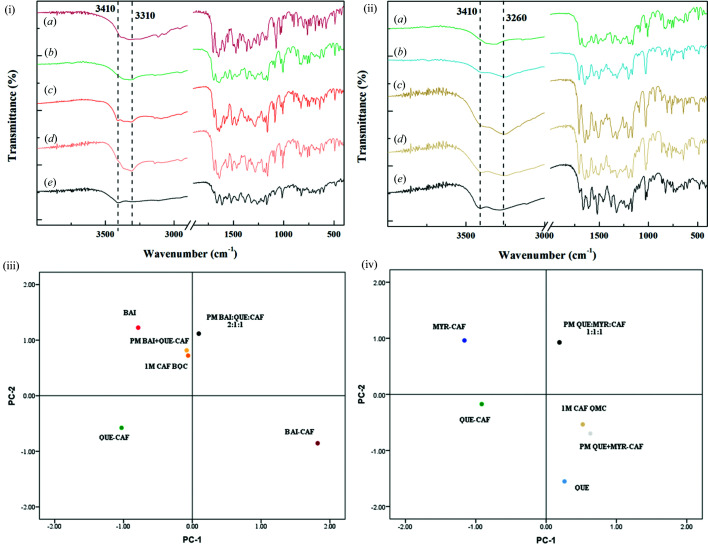
FT-IR spectra of the cocrystals (i)(*a*) BAI–CAF, (i)(*b*) and (ii)(*a*) QUE–CAF, (ii)(*b*) MYR–CAF; systems of (i)(*c*) 1 *M* CAF BQC and (ii)(*c*) 1 *M* CAF QMC prepared by slurry reaction experiments; physical mixtures of (i)(*d*) BAI and QUE–CAF, (i)(*e*) BAI, QUE and 1 *M* CAF, (ii)(*d*) QUE and MYR–CAF, (ii)(*e*) QUE, MYR and 1 *M* CAF. Principal component analysis scores plot of FT-IR data of the (iii) the BQC system and (iv) the QMC system.

The FT-IR spectra of three cocrystals, slurry products of competitive systems, and the physical mixture of crystals and cocrystals are shown in Fig. 5 ▸. In order to explore the potential intermolecular interactions formed in the cocrystal system, the absorption range 3000–3500 cm^−1^ was employed to analyze the potential hydrogen-bond formation between hydroxyl groups of flavonoids with hydrogen acceptors in CAF. In the FT-IR spectra of the BAI, QUE and CAF physical mixture, the absorption at 3410 cm^−1^ could be attributed to the O—H characteristic stretching vibration peak [Fig. 5 ▸(i)(*e*)]. The absorption vibration peaks of O—H moved to 3316 and 3311 cm^−1^ in cocrystals 1 and 2, respectively [Figs. 5 ▸(i)(*a*) and 5(i)(*b*)]. Fig. 5 ▸(i)(*c*) is the FT-IR spectra of BQC mixture. Compared with the crystal physical mixture, the absorption peak of the cocrystal at 3310 cm^−1^ is clearly visible, while the peak at 3410 cm^−1^ also exists, indicating that the BQC system contained both crystal and cocrystal. The O—H stretching vibration peaks shifted from 3410 and 3286 cm^−1^ to 3406 and 3260 cm^−1^ when cocrystal 3 formed [Fig. 5 ▸(ii)(*b*)]. The absorption peaks of the QMC mixture are located at 3410 and 3260 cm^−1^ [Fig. 5 ▸(ii)(*c*)]. The characteristic stretching vibration peak of cocrystal 2 is located at 3310 cm^−1^ [Fig. 5 ▸(ii)(*a*)], which did not appear in the QMC mixture spectra indicating that no cocrystal 2 had been formed.

The absorption peak of the BQC mixture in FT-IR spectra was consistent with the absorption peak of the physical mixture of crystalline BAI and cocrystal 2 (2:1) [Fig. 5 ▸(i)(*d*)] and clearly different from that of the physical mixture of crystalline BAI, QUE and CAF (2:1:1) [Fig. 5 ▸(i)(*e*)]. The principal component analysis (PCA) provided information in agreement with these results. PCA score plot investigations revealed an effective strategy to explore spectral similarities (Mandrile *et al.*, 2019 ▸). Similarities and differences between the samples were determined by principal components (PC) 1 and 2 [Figs. 5 ▸(iii) and 5(iv)]. The cocrystals were located at different quadrants based on certain differences in the composition of the two PCs. The same class samples may have a similar chemical composition (Fan *et al.*, 2013 ▸). By comparing the locations of these samples, it can be learned that the competitive system and the physical mixture of crystal and cocrystal in the same quadrant are located closely in a small area, and the separation of the crystal physical mixture is very obvious. From the results above, only one cocrystal (cocrystal 2 or 3) was formed in the slurry products (BQC or QMC mixture) of the competitive system, which differed from the physical mixture of three crystals.

The PXRD measurement results of crystalline BAI and QUE, competitive systems and the physical mixture of crystal and cocrystal are shown in Fig. 3 ▸. The BQC mixture [Fig. 3 ▸(*f*)] had clear crystal characteristic diffraction peaks at 8.5, 10.1, 10.4, 15.3 and 26.3° 2θ. The diffractogram of the physical mixture of BAI and cocrystal 2 (2:1) [Fig. 3 ▸(*g*)] are the simple superposition of diffraction peaks of crystal BAI and cocrystal 2. The characteristic diffraction peaks of the BQC mixture were consistent with the physical mixture of BAI and cocrystal 2, containing characteristic peaks of the BAI crystal (15.3°) [Fig. 3 ▸(*a*)] and cocrystal 2 (26.3°) [Fig. 3 ▸(*d*)], and no characteristic peaks of cocrystal 1 (3.5°) [Fig. 3 ▸(*c*)]. The PXRD results indicate that, in the BQC mixture, when 1*M* CAF is added, the slurry product was a mixture of BAI crystal and cocrystal 2.

The PXRD diffractograms of the QMC mixture are shown in Figs. 3 ▸(*h*) and 3(*i*). The characteristic diffraction peaks of the physical mixture of QUE and cocrystal 3 [Fig. 3 ▸(*i*)] appeared at 5.3, 10.7, 12.3, 16.1 and 27.2° 2θ, which are identical to the slurry product of the QMC mixture [Fig. 3 ▸(*h*)], indicating that the slurry product of QMC mixture was composed of crystal QUE and cocrystal 3, without cocrystal 2.

### 3.3. Separation performance of flavonoids   

**Figure 6:**
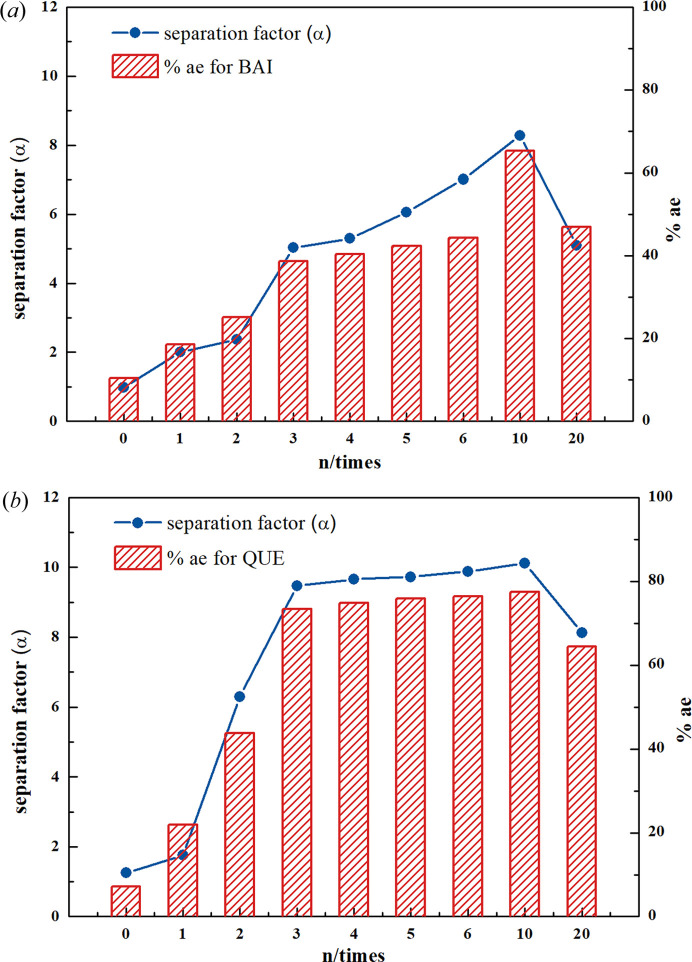
Variations in separation factor and %ae with respect to the amounts of solvent: (*a*) BQC system, (*b*) QMC system.

The separation efficiency was evaluated by two parameters including the separation factor (α) and flavonoid analogs excess value (%ae). The results were shown in Fig. 6 ▸. Different amounts of methanol were added to the slurry products to investigate the effect of solvent amount on the resolution efficiency of flavonoids.

As shown in Fig. 6 ▸, QUE showed stronger separation abilities with MYR [Fig. 6 ▸(*b*)] and BAI [Fig. 6 ▸(*a*)] by cocrystallization with CAF. The separation factor and %ae of BAI and QUE with three times the amount of methanol reached a higher value. Meanwhile, the separation performance was optimized when the volume ratio of methanol and slurry system was 10:1. After adding ten times the amount of methanol, the BAI content was reduced from 61 to 9.76%, and the QUE content was reduced from 51 to 8.49%. Therefore, the separation method can produce >90% purity flavonoid compounds.

### 3.4. Solubility studies of cocrystals   

**Figure 7:**
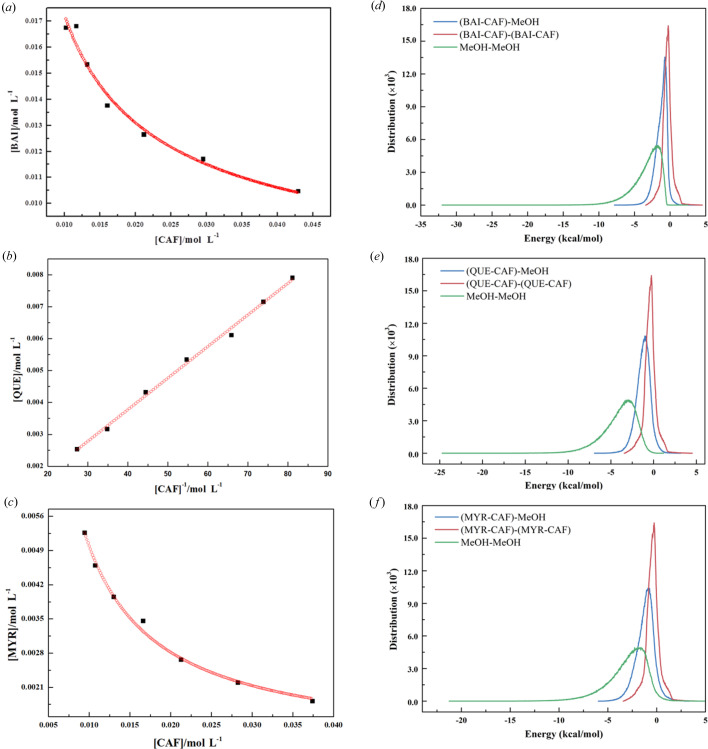
Solubility curve of three cocrystals in methanol at 25°C: (*a*) BAI–CAF, (*b*) QUE–CAF, (*c*) MYR–CAF; and binding energy distributions for the three base-screen pairs (*d*) BAI–CAF, (*e*) QUE–CAF, (*f*) MYR–CAF with methanol.

The mass fraction solubilities of cocrystals were different from those of single components, and their solubilities were calculated using *K*_c_ (Zhang *et al.*, 2007 ▸). The phase solubility profile of CAF to flavonoids in methanol was obtained by stirring an excess of cocrystal in a solution with different concentrations of CAF. As shown in Figs. 7 ▸(*a*) and 7(*c*), the solubilities of BAI and MYR decreased nonlinearly with the increase of CAF concentration, exhibiting typical solution-complexation behavior. According to the equations of complex formation, the solubility product of the cocrystals can be obtained as shown in Table 2 ▸. However, linear regression of the solubility of QUE to the reciprocal of CAF concentration was a straight line passing through the origin [Fig. 6 ▸(*b*)]; it was shown that cocrystal 2 fits the solution-free-complexation model in methanol solution. *K*_c_ of cocrystal 3 was smaller than that of cocrystal 2, indicating that cocrystal 3 was easier to precipitate than cocrystal 2 due to the solubility product theory (Zhang *et al.*, 2007 ▸). Because the ratio of BAI and CAF was different from cocrystals 2 and 3, it was meaningless to compare their solubilities using *K*_c_. As shown in Table 2 ▸, the reaction Δ*G*° of the cocrystals at 25°C was calculated using *K*_c_. From this table, the negative values of Δ*G*° indicated that the three cocrystals were spontaneously generated in methanol at 25°C. Among the three cocrystals, the absolute value Δ*G*° of cocrystal 3 was the highest, indicating that cocrystal 3 would be the first to form spontaneously in the QMC system, which was consistent with the result of the solution convergence model. These data showed that the absolute value Δ*G*° of cocrystal 1 was smaller than that of cocrystal 2, suggesting that cocrystal 2 would be the first to form spontaneously in the BQC mixture in methanol.

The *Blends* module combines the improved Flory–Huggins models with molecular simulation techniques to calculate the compatibility of binary mixtures (Akkermans *et al.*, 2013 ▸). The simulation results of the solubility of the three cocrystals in methanol solution are shown in Figs. 7 ▸(*d*)–7(*f*). The binding energy distribution curve between cocrystal molecules is shown in the red curve of the figure, the binding-energy distribution curve between solvent and molecule is shown in green, while the curve between the cocrystal molecule and the solvent molecule is shown in blue. By combining the degree of similarity between the energy distribution curves, it can be seen that cocrystal 1 had the strongest affinity with methanol, supporting the idea that cocrystal 1 had better solubility in methanol than cocrystals 2 and 3. These molecular simulation results could be correlated with *K*_c_ and Δ*G*°.

### 3.5. Crystal structures of cocrystals   

Single-crystal data of the three cocrystals (Fig. 4 ▸) showed that there were O—H⋯O and O—H⋯N hydrogen-bonding interactions (selected hydrogen bonds are listed in Table 3 ▸). In order to gain insight into the interactions between the cocrystal constituents (especially hydrogen-bonding inter­actions), Hirshfeld surfaces and their 2D fingerprint plots (Fig. S4) were used to extend the qualitative and quantitative analysis of the interactions (Mackenzie *et al.*, 2017 ▸).

The Hirshfeld surfaces of BAI-I, BAI-II, QUE and MYR are illustrated in Figs. S4(*a*)–S4(*d*), showing surfaces mapped with *d*_norm_. Analysis of the interactions in cocrystal 1 showed that there was a strong O—H⋯N hydrogen bond between the nitrogen atom in the CAF and the BAI-II molecular hydroxyl group (Table 3 ▸). Comparing the fingerprint plots of the H⋯N contacts in Figs. S4(*a*) and S4(*b*), it was found that these H⋯N contacts accounted for 2.3% of the total *d*_norm_ surface of BAI-II, whereas BAI-I had almost no H⋯N contacts with CAF [Fig. S4(*e*)]. Moreover, these interactions are shown as spikes [Fig. S4(*b*)] on the 2D fingerprint map of the upper (donor) region, whereas the the lower (receptor) region had no peaks, indicating that only the BAI-II hydroxyl groups could be used as hydrogen-bond donors to form a strong O—H⋯N hydrogen bond in cocrystal 1. The hydroxyl groups of QUE and MYR are simultaneously engaged in O—H⋯N interactions with the nitrogen atom of the CAF molecule, and both showed sharp spikes in the upper (donor) regions of the fingerprint plot of QUE and MYR [Figs. S4(*c*) and S4(*d*)]. The contribution value of O—H⋯N, a strong hydrogen bond, in cocrystal 3 interactions (2.7%) was similar to the contribution value in cocrystal 2 (2.4%) [Fig. S4(*e*)].

The longer and thinner spikes on the 2D fingerprint map reflected the H⋯O contacts of the hydrogen bond O—H⋯O (Soman *et al.*, 2014 ▸; Sowa *et al.*, 2013 ▸). Furthermore, in the upper (donor) regions of the fingerprint plot, the H⋯O spike was essentially longer than the above-mentioned H⋯N spike, indicating that the H⋯N distance for the O—H⋯N was longer than the H⋯O distance for O—H⋯O (Fig. S4; O⋯H/H⋯O and N⋯H/H⋯N). Thus, the hydrogen bond O—H⋯O formed by the hydroxyl group as a hydrogen donor of BAI-I, BAI-II, QUE and MYR also played a key role in guiding the formation of cocrystals (the contribution of the H⋯O contact was 10.0, 7.9, 9.1 and 11.5%, the contribution of the O⋯H contact was 13.6, 13.8, 22.1 and 22.6%, respectively). For the BAI-II molecule, the O⋯H spike was shorter than other molecules on the 2D fingerprint map of the lower (receptor) region, indicating longer H⋯A distances for the O—H⋯O bonding of BAI-II compared with other molecules (Fig. S4 O⋯H/H⋯O). In total, the H⋯O/O⋯H and H⋯N interactions contribute to 23.7, 24.0, 33.6 and 36.8% of the total surface of the BAI-I, BAI-II, QUE and MYR molecules, respectively. In addition, a number of other weak contacts (including H⋯C/C⋯H and H⋯H) constitute the majority of the *d*_norm_ surfaces, most of which were atomic contacts between the same molecules.

Hirshfeld surface analysis allowed us to visually inspect the electron density configuration of the three flavonoids. Fig. S4 shows the interaction fingerprints of the different flavonoid molecules on the surface. The H⋯O and H⋯N contacts are in a tight contact region, indicating that two strong hydrogen bonds, O—H⋯N and O—H⋯O, are formed, and the N and O atoms on the surface of the CAF molecule play an important role in the cocrystal formation.

**Figure 8:**
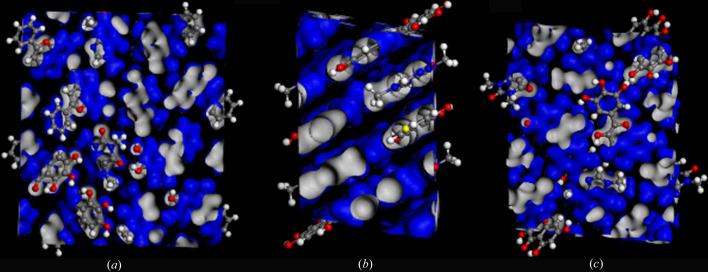
Electron density of three different cocrystals (*a*) BAI–CAF, (*b*) QUE–CAF, (*c*) MYR–CAF.

Electronic structure analysis based on the DOS illustrates the nature of the bond between the molecular crystals (Feng *et al.*, 2017 ▸). The total DOS of the three cocrystals was shown in Fig. S5, the majority of the peaks produced by cocrystals 2 and 3 were obviously below the Fermi level, indicating that their orbits were almost full (An *et al.*, 2008 ▸; Reshak *et al.*, 2013 ▸). As shown in Figs. 8 ▸(*a*)–8(*c*), electron density (blue area) reflected the strength of bonding between two adjacent atoms. The greater the electron-density overlap, the stronger the bonding of the two atoms will be. From the electron density of each cocrystal unit cell, it can be found that the electron clouds of cocrystal 1 partially overlap, cocrystal 2 has strong overlap in the two-dimensional structure, while the electron density was far away in the three-dimensional structure, and the electron density of cocrystal 3 overlaps most, indicating that cocrystal 3 has the strongest neighboring atomic bonding and the bonding of cocrystal 1 was the weakest.

The PDOS of the N1 and O2 atoms (Fig. S9) in the CAF molecule and related H atoms were shown in Figs. S6 and S7 to further analyze detailed electron overlap between atoms. It can be learned from PDOS that the electron densities of all energy states were lowered with the peaks distributed from 0 to −0.9 Ha after forming the cocrystal. The overlap of PDOS verified the formation of bonds between atomic pairs (Guo *et al.*, 2011 ▸). From the degree of overlap, the H(*s*) and N(*p*) orbits were ordered cocrystal 3 > cocrystal 2 > cocrystal 1 (in 0 to −0.2, −0.2 to −0.4 and −0.8 Ha). Moreover, there were more overlaps between the O(*p*) orbital and the H(*s*) orbital in cocrystals 2 and 3 than in cocrystal 1 (between −0.2 and −0.5 Ha). In particular, the O(*p*) orbital and H(*s*) orbital in −0.2 to −0.3 Ha almost completely overlapped in cocrystal 3. It was shown that, in the process of forming hydrogen bonds, the close connection of atoms in cocrystal 3 was stronger than that of cocrystal 2, followed by cocrystal 1.

## 4. Discussion   

In this study, three flavonoid cocrystals (*i.e.* cocrystals 1, 2 and 3) were prepared and characterized. Among them, a single crystal of cocrystal 3 was obtained for the first time by the solvent slow evaporation method, the single-crystal data (shown in Table 1 ▸) were deposited in the CCDC (reference No. 1964231). Additionally, the selected CAF and three structurally similar flavonoids were used as model drugs to investigate the cocrystal formation behavior in ternary systems. Surprisingly, the flavonoids formed cocrystals with CAF competitively in a ternary system. In order to explore the internal mechanism of such competitive cocrystallization behavior, solubility product analysis, thermodynamics of cocrystal formation, computer modeling of intermolecular interactions by *Blends* analysis, Hirshfeld surface analysis and DOS were performed.

It was found that MYR could form a 1:1 complex with CAF in methanol solution whereas QUE could not, the *K*_c_ value of cocrystal 3 was 2.5-fold lower than cocrystal 2, indicating that CAF would preferentially form a cocrystal with MYR rather than QUE in methanol solution and potentially achieve the separation of these two flavonoids. As a 2:1 cocrystal, the solubility of cocrystal 1 cannot be directly compared with the 1:1 cocrystal through the value of *K*_c_. Just like the inorganic salts AgCl and Ag_2_Cr_2_O_7_, although the *K*_sp_ of Ag_2_Cr_2_O_7_ (2.0 × 10^−12^) is smaller than that of AgCl (1.8 × 10^−10^), Ag_2_Cr_2_O_7_ was more soluble than AgCl in the same solvent system. Based on thermodynamic calculations, the nucleation Δ*G*° values of all three cocrystals were negative in the order cocrystal 1 > cocrystal 2 > cocrystal 3, suggesting that cocrystal 1 was more difficult to form than the other two cocrystals.

We then simulated the interaction between the cocrystal and solvent molecules through the *Blends* module to analyze the compatibility of different cocrystal molecules with solvents. The result showed that the cocrystal solubility order in methanol was cocrystal 1 > cocrystal 2 > cocrystal 3. Using the *Blends* module, we tried to simulate the affinity of flavonoids with methanol and the CAF molecule (Fig. S8 and Table S1 of the supporting information). From the overlap of energy distribution curves, the affinity of flavonoids with CAF was stronger than that of methanol, and the larger overlap area between each cocrystal corresponded to more tightly bound complexes. The overlap area of cocrystal 3 [Fig. S8(*c*)] was significantly higher than for cocrystal 2 [Fig. S8(*b*)] and cocrystal 1 [Fig. S8(*a*)], indicating that the affinity of MYR for CAF was stronger than that for QUE and BAI. The formation process of cocrystals could be regarded as the process of competitively binding flavonoids with methanol and CAF, then all three flavonoids tend to combine with CAF molecules, and the strength of binding is cocrystal 3 > cocrystal 2 > cocrystal 1.

Hydrogen bonding is one of the most important interactions in the process of cocrystal formation. The Hirshfeld surface is unique for a given crystal structure, indicating that it is possible to gain a better understanding of the crystal (Spackman & Jayatilaka, 2009 ▸). After Hirshfeld surface analysis for hydrogen-bonding interactions based on single-crystal structures of three flavonoid cocrystals, it was found that O—H⋯N and O—H⋯O, acting as two strong hydrogen-bonding forces, formed interactions between the flavonoids and CAF molecules more easily. The more the strong interactions contribute [MYR 36.8% > QUE 33.6% > BAI-II 24.0% > BAI-I 23.7%, Fig. S4(*e*)], the easier the formation of a cocrystal will be (Soman *et al.*, 2014 ▸), suggesting MYR would form a cocrystal much easier with CAF than the other two flavonoids.

The ability to form tight interactions in a finite space is also the key to cocrystal formation. DOS can help us to visually understand the electron density of cocrystal molecules, and the bond between atoms can be judged by the overlap of electron clouds. It is well known that the energy of the atoms in a coformer will significantly decrease and its electron orbitals will be filled by the host compound. Compared with cocrystals 2 and 1, the internal and external electronic cloud connections of cocrystal 3 molecules were the closest (Figs. S5 and S6). Cocrystal 1 still had many empty electron orbitals, and the degree of overlap of electronic clouds was relatively poor. The order of greatest electronic cloud overlap was cocrystal 3, 2 and then 1, which was consistent with the solubility product analysis (Table 2 ▸) and Hirshfeld surface analysis (Fig. S4).

The packing of cocrystals plays an equally important role in crystal formation alongside hydrogen bonding. Energy calculations can provide the relative importance of various interactions between molecules in cocrystals. The minimum value of the HOMO–LUMO energy gap (Δ*E*) of the complex molecular system reflects that it is more polarizable (Jyothi & Lokanath, 2019 ▸). Hence, we observed that cocrystal 3 was more stable than cocrystals 2 and 1 (Fig. S11). These results could be correlated with the intermolecular interactions detected in the crystal structure (Fig. 4 ▸).

In order to investigate the reactivity of competitive cocrystallization, 2 *M* CAF was added to the slurry containing BAI and QUE (or QUE and MYR) for reaction. Different from the 1 *M* CAF BQC system, the characteristic diffraction peaks of BAI (15.3°) in the 2 *M* CAF BQC system [Fig. S10(*h*)] disappeared, which was replaced by characteristic diffraction peaks of cocrystal 1 (3.5°), indicating that no BAI crystals remained during the slurry process; meanwhile cocrystal 1 was formed. It was suggested that when 1 *M* CAF is added to methanol solution containing 1 *M* BAI and 1 *M* QUE, only cocrystal 2 is formed, whereas adding 2 *M* CAF produces both cocrystal 1 and cocrystal 2. Such a phenomenon indicated that BAI and QUE could be separated by controlling the amount of the coformer CAF added via competitive cocrystallization. Based on our separation experiments (Fig. 6 ▸), the two flavonoids with similar chemical structures (*i.e.* BAI and QUE/QUE and MYR) could be separated from each other with 90% efficiency in a single competitive cocrystallization process and the yield is greater than 80% (for BAI and QUE, Fig. S12). To verify the feasibility of the cocrystallization separation techniques, separation of BAI and MYR and three flavonoids (BAI, QUE, MYR) was considered. Com­petitive priority was given to the formation of cocrystal 3 and the purity of MYR was improved by precipitation (from 37.20 to 81.59% in BAI, MYR, from 26.66 to 57.75% in BAI, QUE, MYR, Fig. S13). The above results show that such a process provided a green chemical separation for flavonoids with fewer steps and lower energy consumption than the traditional organic solvent extraction method, preparation liquid chromatography and capillary electrophoresis (Pyo *et al.*, 2009 ▸; Liu *et al.*, 2014 ▸; Wu *et al.*, 2018*b* ▸).

## 5. Conclusions   

In the present study, cocrystals show a distinct competitive order of similar structure flavonoids when the competing coformer CAF is offered. In addition, the formation of the final product can be predicted by calculating the formation constant and Gibbs free energy of the possible reaction cocrystal. Quantitative assessment of the single-crystal structure revealed diverse contributions and strengths of intermolecular interactions in different cocrystals from Hirshfeld surfaces, DOS analysis and energy calculation, directly indicating different reactivity. Such a method can be useful for flavonoid separation: CAF was added to the BAI–QUE (or QUE–MYR) mixture and competitively formed cocrystal 2 (or cocrystal 3), using the solid–liquid separation method, to obtain a high-purity (>90%) flavonoid. The proposed method explores a new path for green and efficient separation of similar-structure natural products.

## Supplementary Material

Crystal structure: contains datablock(s) I. DOI: 10.1107/S2052252520015997/yc5023sup1.cif

Supporting information. DOI: 10.1107/S2052252520015997/yc5023sup2.pdf

CCDC reference: 1964231

## Figures and Tables

**Table 1:** Crystallographic data for three cocrystals

	BAI–CAF†	QUE–CAFMeOH†	MYR–CAF‡
Formula	C_38_H_30_O_12_N_4_	C_24_H_24_O_10_N_4_	C_23_H_20_O_10_N_4_
Crystal system	Monoclinic	Monoclinic	Monoclinic
CCDC No.	1522878	1428198	1964231
Space group	*P*2_1_/*c*	*P*2_1_/*c*	*P*2_1_/*n*
Temperature (K)	100 (2)	100 (2)	293 (2)
*a* (Å)	24.669 (3)	10.309 (3)	19.932 (4)
*b* (Å)	4.5593 (5)	14.853 (4)	4.4610 (9)
*c* (Å)	28.267 (3)	15.199 (5)	24.275 (5)
α (°)	90	90	90
β (°)	90.320 (8)	100.612 (2)	97.73 (3)
γ (°)	90	90	90
*V* (Å^3^)	3179.2 (6)	2287.51 (12)	2138.8 (7)
*D*_Cal_ (g cm^−3^)	1.535	1.535	1.591
*Z*	4	4	4
Independent reflns	5590	4031	3937
*S*	0.954	1.035	1.002
*R*_int_	0.1624	0.0637	0.0856
*R*_1_	0.0586	0.0434	0.0683
*wR*_2_	0.1444	0.1090	0.1569

† Obtained from the literature and the crystallographic data were downloaded from the CCDC.

‡ Prepared in our laboratory.

**Table 2:** Cocrystal formation constant (*K*_c_), complexation models and Gibbs free energy (Δ*G*°) of the three cocrystals

	*K*_c_	Complexation models	*R* ^2^	Δ*G*° (kJ mol^−1^)
BAI–CAF	2.83 × 10^−6^	1:1, 2:1	0.9654	−2.483
QUE–CAF	9.59 × 10^−5^	None	0.9951	−6.547
MYR–CAF	3.82 × 10^−5^	1:1	0.9929	−12.004

**Table 3:** Selected hydrogen-bond distances and parameters for the cocrystals

Cocrystal	Interaction	D—H (Å)	H⋯A (Å)	D⋯A (Å)	∠D—H⋯A (°)
BAI–CAF	O_10_—H_10_⋯N_2_	0.82	2.02	2.78	154.9
	O_3_—H_3_⋯O_2_	0.91	1.76	2.60	151.6
	O_4_—H_4_⋯O_11_	0.89	1.98	2.82	158.0
	O_5_—H_5_⋯O_12_	0.95	1.84	2.71	149.6
	O_8_—H_8_⋯O_7_	0.91	1.72	2.59	157.1
	O_9_—H_9_⋯O_2_	0.89	1.80	2.64	154.9
QUE–CAF	O_2_—H_2_⋯N_21_	0.84	2.04	2.82	154.8
	O_2_—H_2_⋯O_3_	0.84	2.26	2.70	113.6
	O_5_—H_5_⋯O_12_	0.84	2.43	2.84	111.4
	O_5_—H_5_⋯O_3_	0.84	1.85	2.59	148.4
	O_7_—H_7_⋯O_22_	0.84	1.88	2.71	173.1
	O_13_—H_13_⋯O_5_	0.84	1.96	2.77	163.5
	O_12_—H_12_⋯O_31_	0.84	1.79	2.62	174.9
	O_13_—H_13_⋯O_12_	0.84	2.31	2.75	113.4
	O_31_—H_31_⋯O_21_	0.84	1.88	2.71	169.4
MYR–CAF	O_6_—H_6_⋯N_1_	0.82	3.19	3.64	116.8
	O_2_—H_2_⋯N_2_	0.82	2.04	2.79	152.3
	O_6_—H_6_⋯O_5_	0.82	1.86	2.61	150.0
	O_7_—H_7_⋯O_10_	0.82	4.77	5.11	110.3
	O_3_—H_3_⋯O_5_	0.89	1.90	2.75	157.4
	O_4_—H_4_⋯O_5_	0.86	2.10	2.69	125.1
	O_2_—H_2_⋯O_3_	0.82	2.36	2.78	112.7
	O_4_—H_4_⋯O_3_	0.86	2.88	3.35	118.0
