# Supplementary material for: Competitive cocrystallization and its application in the separation of flavonoids
Source: IUCrJ. 2021 Jan 21;8(Pt 2):195–207. doi: 10.1107/S2052252520015997 (PMC7924225; doi:10.1107/S2052252520015997)
Supplement: Supplementary file 2 [file m-08-00195-sup2.pdf]

IUCrJ

Volume 8 (2021)

Supporting information for article:

Competitive cocrystallization and application in separation of flavonoids

Yanming Xia, Yuanfeng Wei, Hui Chen, Shuai Qian, Jianjun Zhang and Yuan Gao

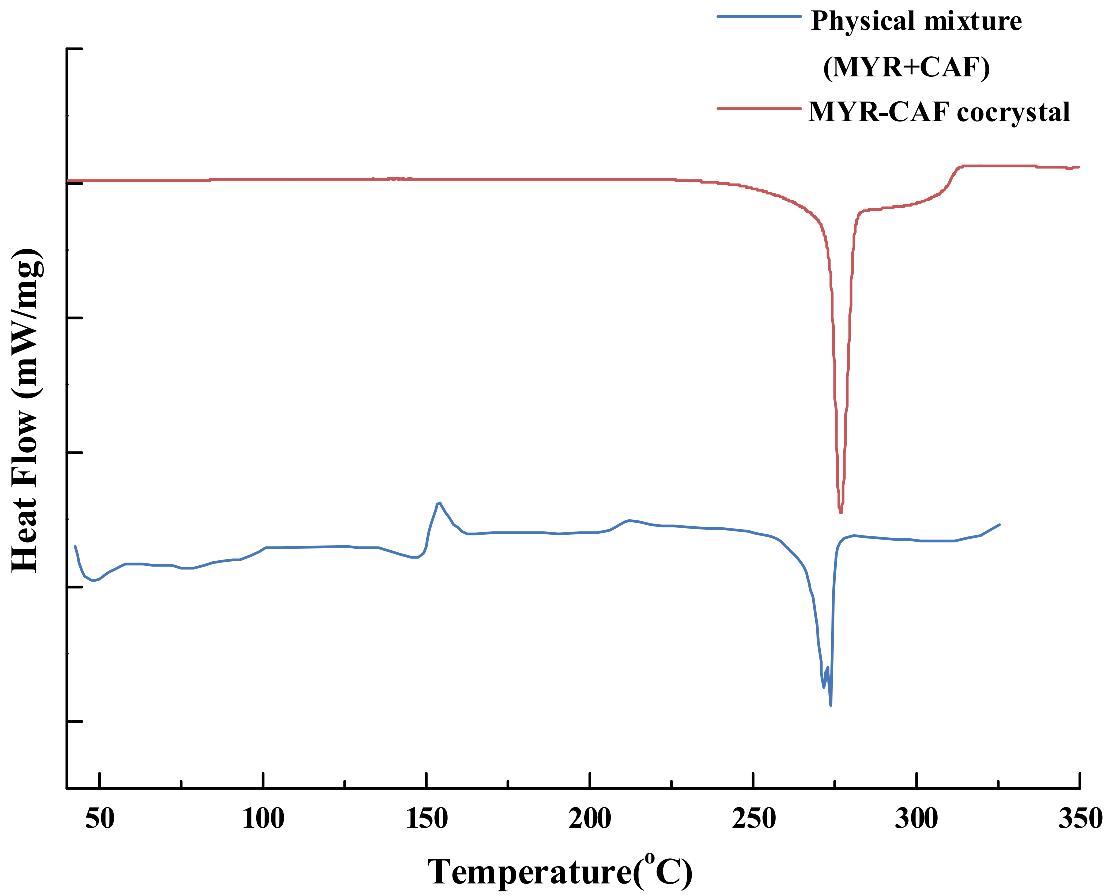

Figure S1 DSC profiles of MYR-CAF cocrystal and physical mixture of MYR and CAF.

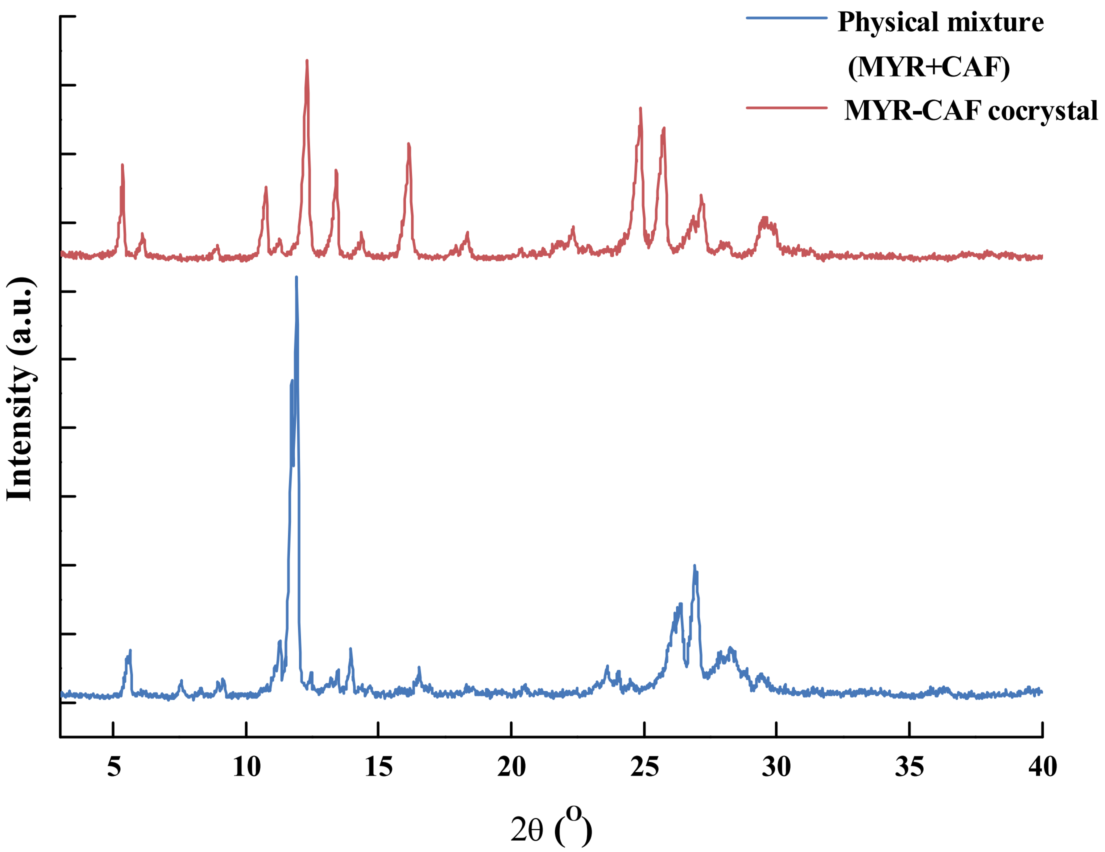

Figure S2 PXRD result of MYR-CAF cocrystal and physical mixture of MYR and CAF.

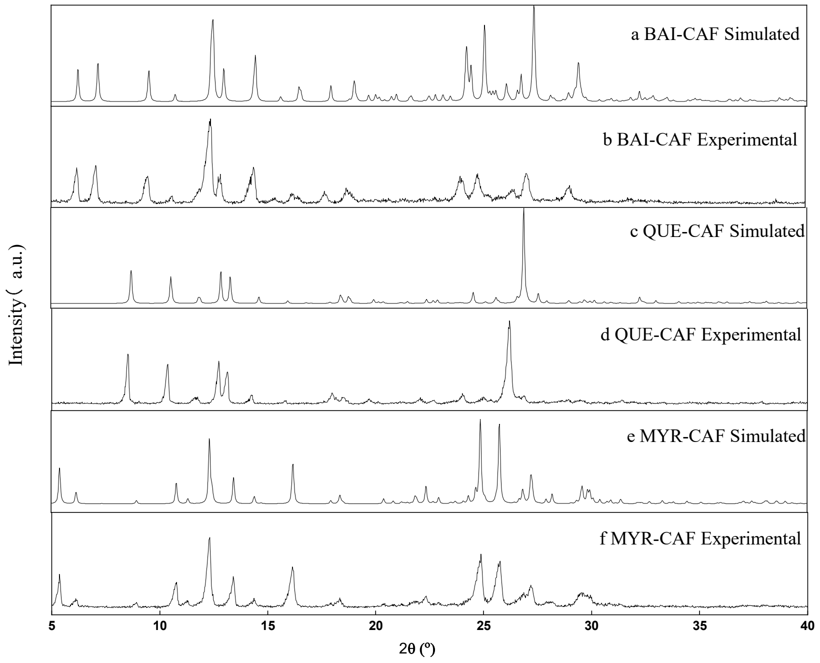

Figure S3 Powder XRD patterns of BAI-CAF (simulated) (a), BAI-CAF (experimental) (b), QUE-CAF (simulated) (c), QUE-CAF (experimental) (d), MYR-CAF (simulated) (e), MYR-CAF (experimental) (f).

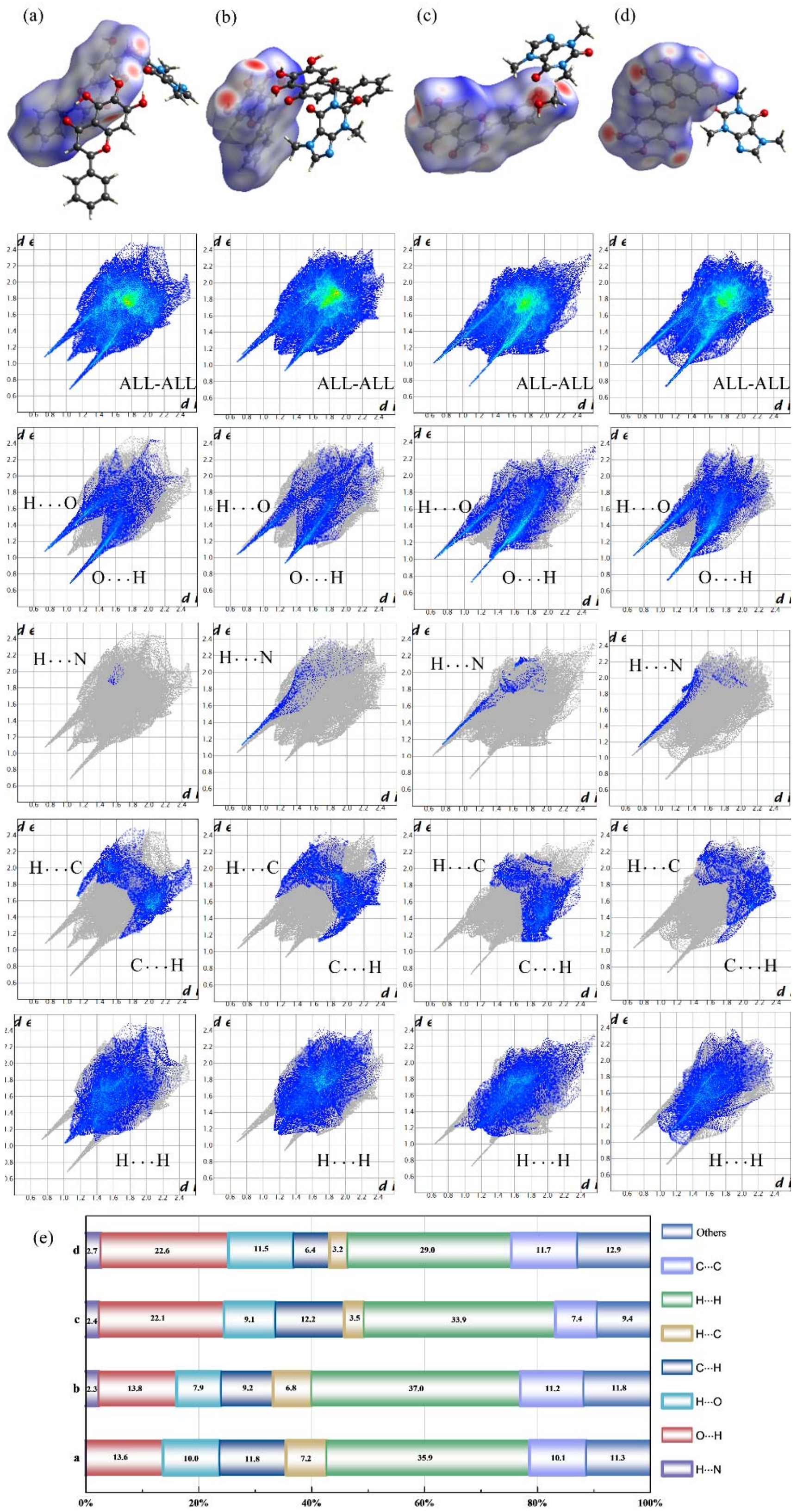

Figure S4 Hirshfeld surfaces mapped with d_{norm} and 2D finger print plots with d_i and d_e (a, BAI-I, b, BAI-II, c, QUE, d, MYR), and (e) the distribution of intermolecular interactions (a, BAI-I, b, BAI-II, c, QUE, d, MYR).

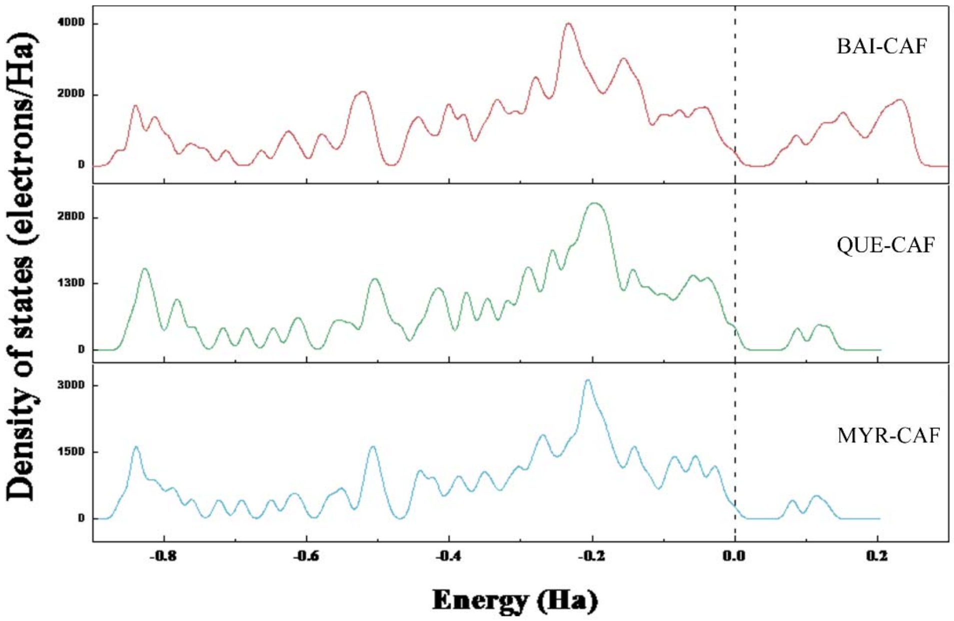

Figure S5 The total DOS (a) of three different cocrystals (the dotted line represents the Fermi level).

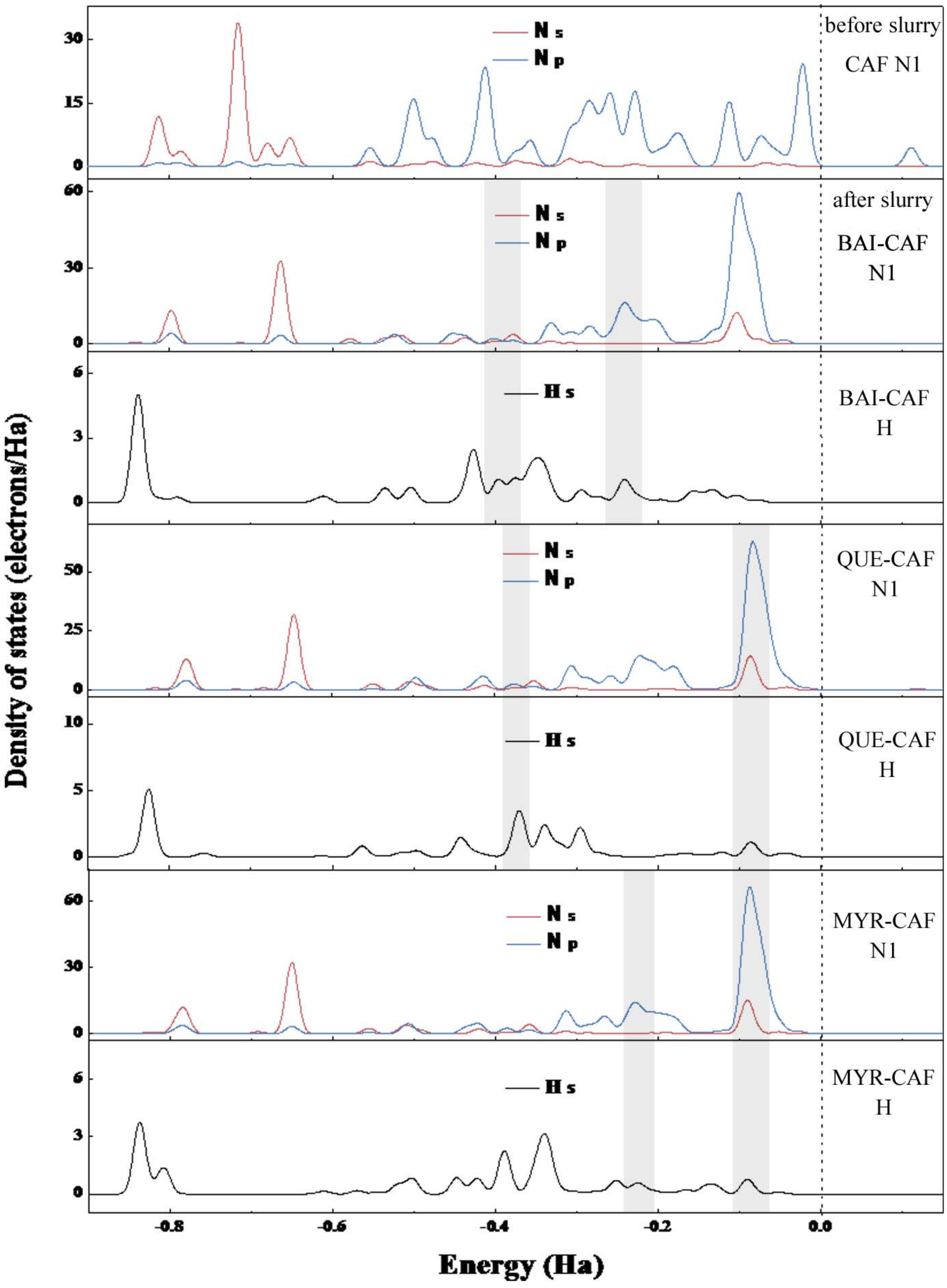

Figure S6 PDOS of N1 atom on CAF before and after slurry and the hydrogen bond linked H atoms on the surface of different cocrystals (the dotted line represents the Fermi level).

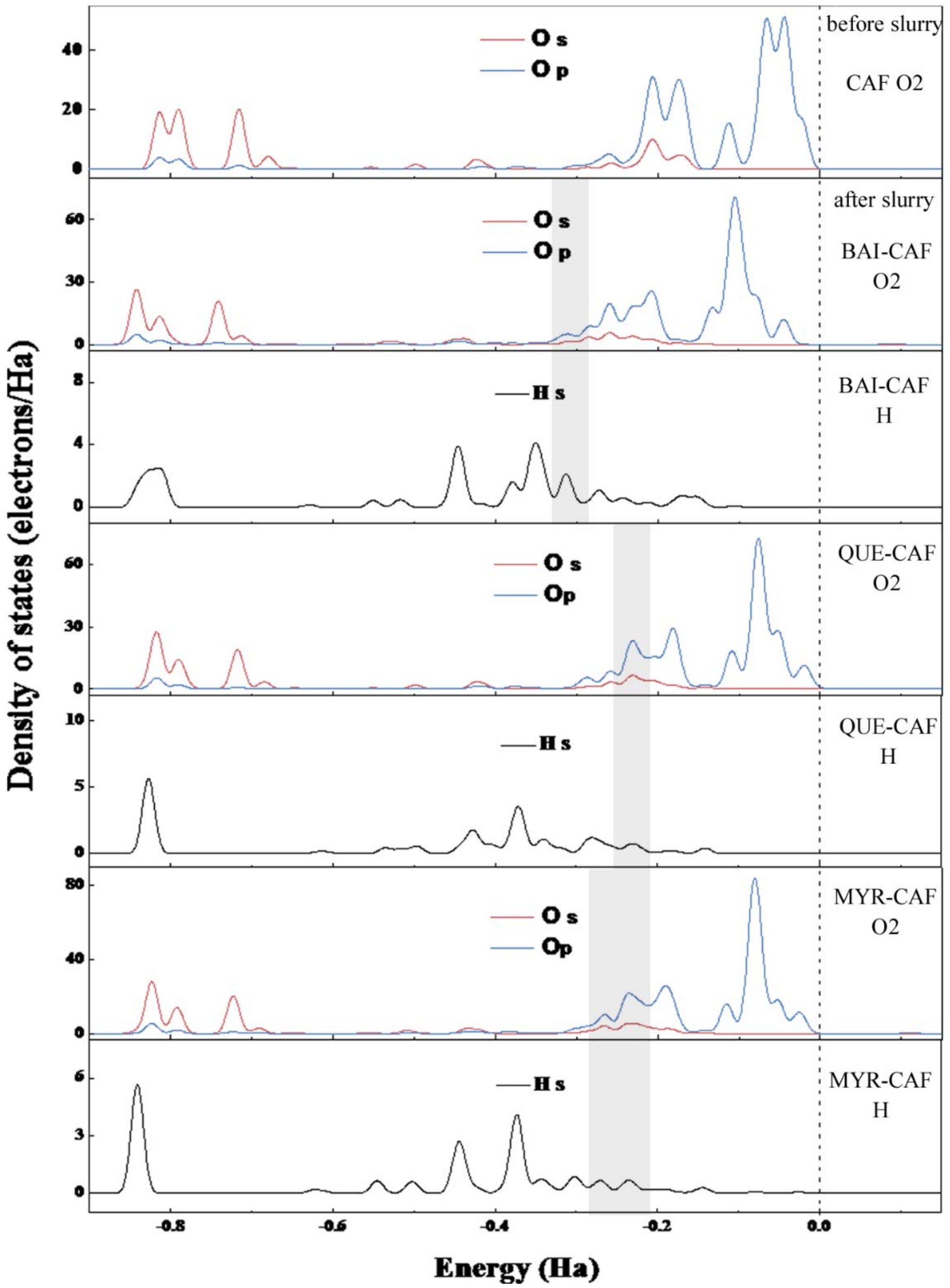

Figure S7 PDOS of O2 atom on CAF before and after slurry and the hydrogen bond linked H atoms on the surface of different cocrystals (the dotted line represents the Fermi level).

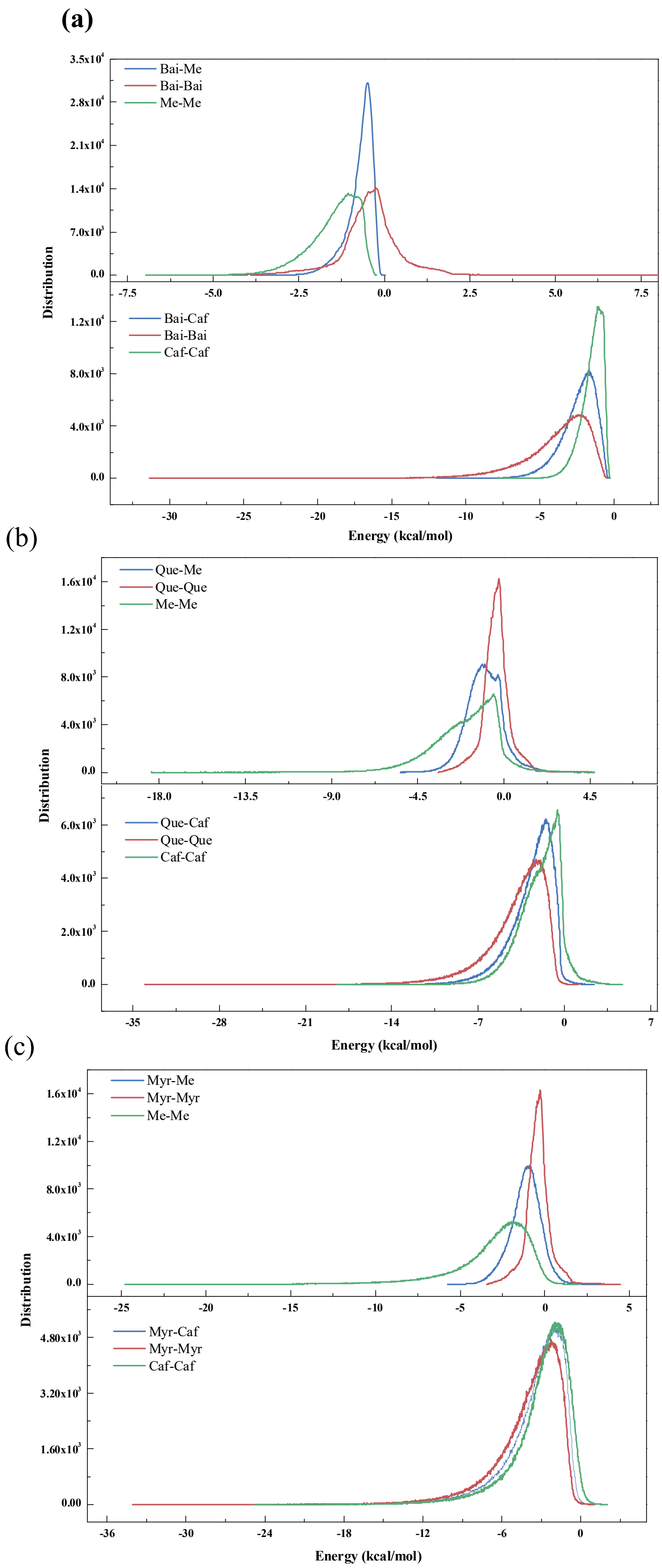

Figure S8 Binding energy distributions for the three base-screen pairs (a, BAI, b, QUE, c, MYR with CAF).

Table S1 Blends study table output

Base	Screen	Chi(298K)	E_{mix}(298K)	E_{bb} avg(298K)	E_{bs} avg(298K)	E_{ss} avg(298K)
MeOH	BAI	13.05	7.72	-2.66	-1.19	-3.45
CAF	BAI	82.00	48.56	-30.79	-9.76	-3.45
MeOH	QUE	61.34	36.32	-1.57	-2.63	-15.01
CAF	QUE	73.63	43.60	-33.88	-16.25	-15.01
MeOH	MYR	86.05	50.96	-1.57	-2.84	-24.45
CAF	MYR	23.78	14.08	-34.00	-25.20	-24.45

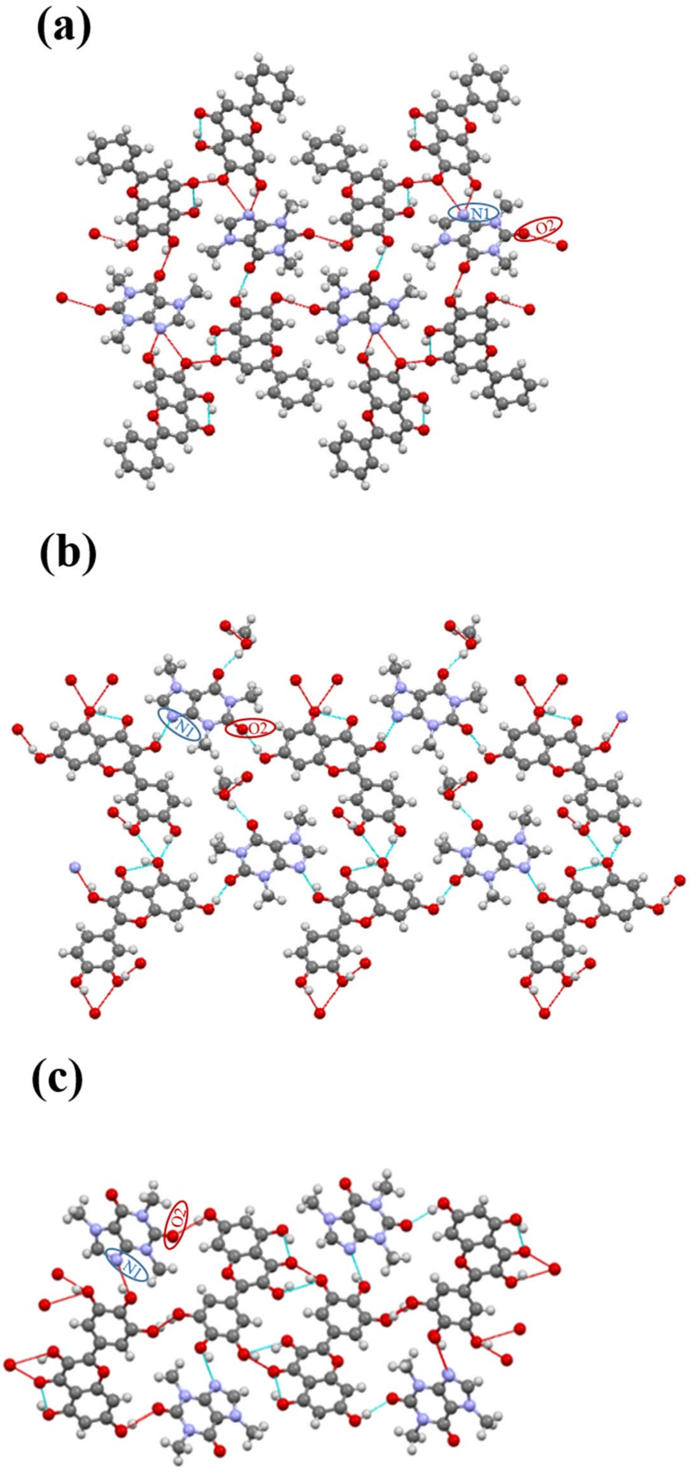

Figure S9 Schematic packing motif of cocrystals (a, BAI-CAF b, QUE-CAF c, MYR-CAF) with hydrogen bonds (hydrogen bonds between molecules forming the heteromolecular dimer are indicated by red lines, and blue dashed lines represent the inter and intra hydrogen bonds of the flavonoids molecular).

Table S2 The properties associated with the energy gap

	BAI-CAF	QUE-CAF	MYR-CAF
Ionization Energy	5.76 eV	5.46 eV	5.71 eV
Electron Affinity	2.27 eV	1.87 eV	1.99 eV
Electronegativity	4.01 eV	3.66 eV	3.85 eV
Chemical Potential	-4.01 eV	-3.66 eV	-3.85 eV
Hardness	1.74 eV	1.79 eV	1.86 eV
Softness	0.57 eV	0.55 eV	0.53 eV
Electrophilicity Index	4.62 eV	3.74 eV	3.98 eV

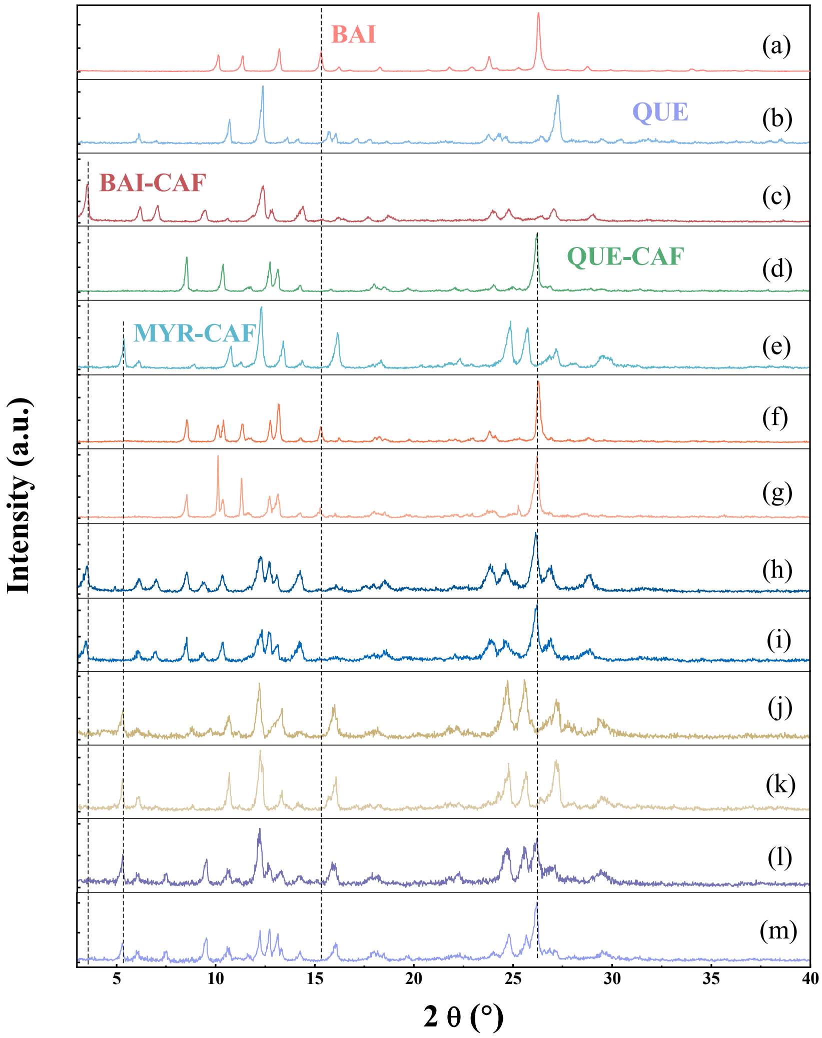

Figure S10 PXRD diffractogram of crystal BAI (a) and QUE (b), slurry products of three cocrystals ((c) BAI-CAF, (d) QUE-CAF, (e) MYR-CAF) and ternary systems ((f) 1M CAF BQC, (h) 2M CAF BQC, (j) 1M CAF QMC, (l) 2M CAF QMC), physical mixture of crystal and cocrystal ((g) BAI: QUE-CAF 1:1, (k) QUE: MYR-CAF 1:1), and physical mixture of two cocrystals ((i) BAI-CAF: QUE-CAF 1:1, (m) QUE-CAF: MYR-CAF 1:1).

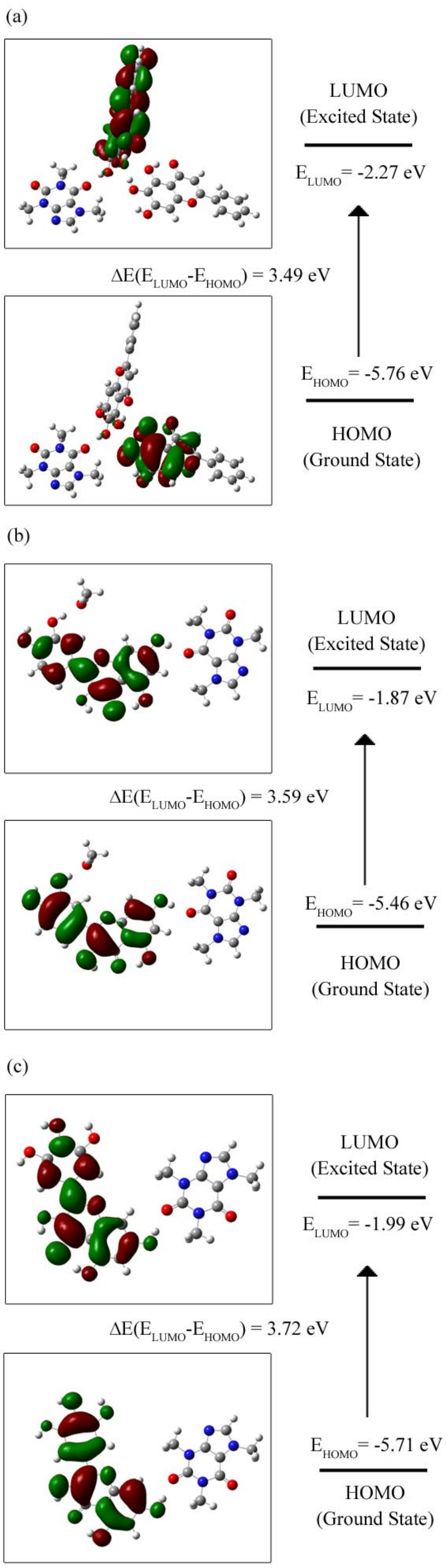

Figure S11 Frontier molecular orbitals of three different cocrystals (a, BAI-CAF, b, QUE-CAF, c, MYR-CAF).

S1. Energy Calculations

The computed frontier molecular orbitals are shown in Figure S11. HOMO orbitals of cocrystal 1 are found over the 4-Oxo-4H-1-benzopyran ring of BAI-II, whereas LUMO orbitals are spread over the BAI-I. Similarly, LUMO orbitals of QUE-CAF and MYR-CAF are spread over the 2-Phenyl-γ-benzopyrone ring of QUE and MYR, respectively, whereas HOMO orbitals fully surround the QUE and MYR, leaving the CAF molecule. These observed features clearly indicated the presence of intramolecular charge transfer within the cocrystal molecules. The chemical stability could be predicted by calculating the energy values of HOMO and LUMO (Li *et al.*, 2020). The energy gap between HOMO and LUMO for cocrystal 1, 2 and 3 can be calculated as 3.49, 3.59 and 3.72 eV, respectively. The sequence of stability among the predictors is cocrystal 3 > cocrystal 2 > cocrystal 1.

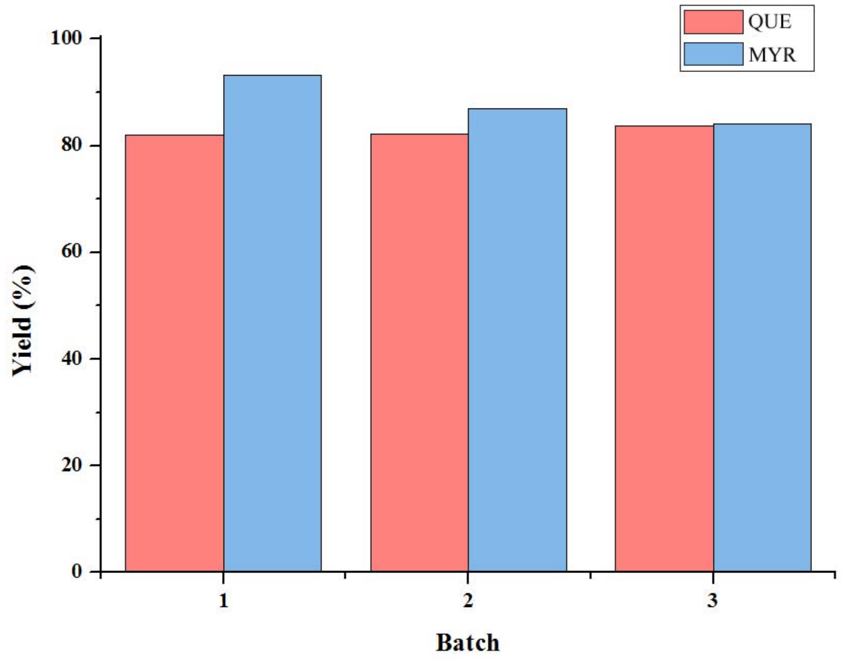

$$\text{Yield of flavonoids } = \frac{\text{The amount of flavonoids in the precipitation (in cocrystal)}}{\text{The content of flavonoids in the mixture (before slurry)}} \times 100\%$$

Figure S12 The yield of QUE (or MYR) in the separation of BAI-QUE (or QUE-MYR).

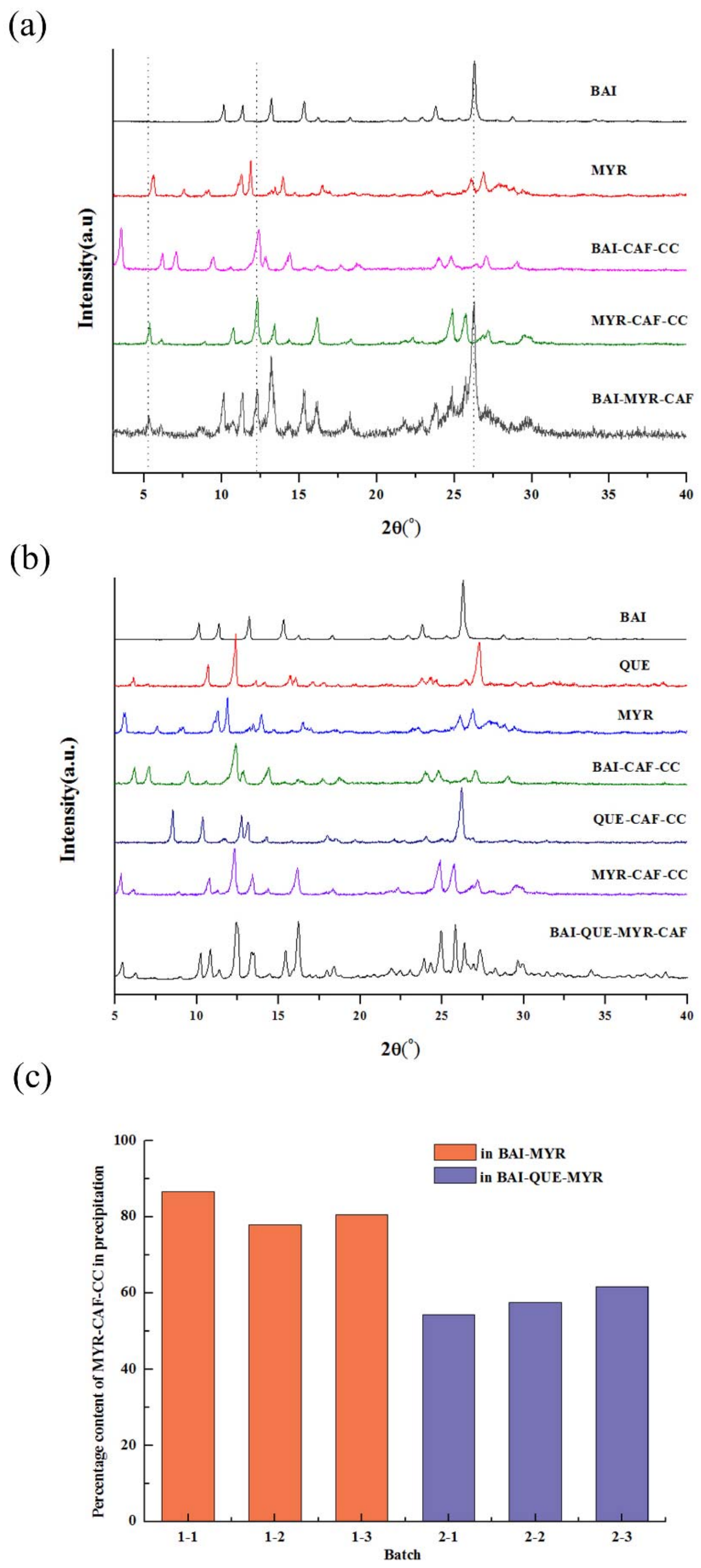

Figure S13 The results of the separation of BAI-MYR and BAI-QUE-MYR

The PXRD diffractograms of BAI-MYR and BAI-QUE-MYR system slurry products were shown in Figure S13a and b. The results indicating that the slurry product of BAI-MYR system is composed of crystal BAI and cocrystal 3, without cocrystal 1. Also in BAI-QUE-MYR system, competitive priority is given to the formation of the cocrystal 3 (MYR-CAF-CC). The separation results Figure S13c also show that the purity of MYR is improved to varying degrees in precipitation (from 37.20% to 81.59% in BAI-MYR, from 26.66% to 57.75% in BAI-QUE-MYR).

Table S3 Selected bond lengths and bond angles for MYR-CAF cocrystals

Atom 1	Atom 2	d 1,2 [nm]	Atom 3	d 1,3 [nm]	Angle 2,1,3
O1	H1A	0.08206	C2	0.13848	109.485
O1	H1A	0.08206	H1A	0.21219	98.571
O1	H1A	0.08206	C1	0.23866	101.745
O1	H1A	0.08206	C3	0.23871	112.327
O1	C2	0.13848	H1A	0.21219	131.394
O1	C2	0.13848	C1	0.23866	30.285
O1	C2	0.13848	C3	0.23871	30.497
O1	H1A	0.21219	C1	0.23866	157.449
O1	H1A	0.21219	C3	0.23871	102.289
O1	C1	0.23866	C3	0.23871	60.78
C1	H1B	0.09298	C2	0.13805	120.398
C1	H1B	0.09298	C6	0.13975	120.367
C1	H1B	0.09298	O1	0.23866	90.011
C1	H1B	0.09298	C3	0.2415	149.628
C1	H1B	0.09298	C5	0.2416	150.765
C1	H1B	0.09298	C7	0.24912	89.717
C1	C2	0.13805	C6	0.13975	119.235
C1	C2	0.13805	O1	0.23866	30.391
C1	C2	0.13805	C3	0.2415	29.23
C1	C2	0.13805	C5	0.2416	88.829
C1	C2	0.13805	C7	0.24912	149.872

C1	C6	0.13975	O1	0.23866	149.618
C1	C6	0.13975	C3	0.2415	90.005
C1	C6	0.13975	C5	0.2416	30.414
C1	C6	0.13975	C7	0.24912	30.662
C1	O1	0.23866	C3	0.2415	59.619
C1	O1	0.23866	C5	0.2416	119.219
C1	O1	0.23866	C7	0.24912	178.513
C1	C3	0.2415	C5	0.2416	59.6
C1	C3	0.2415	C7	0.24912	120.652
C1	C5	0.2416	C7	0.24912	61.076
O2	H2A	0.08195	C3	0.13587	109.431
O2	H2A	0.08195	C2	0.23391	141.292
O2	H2A	0.08195	C4	0.24221	81.282
O2	H2A	0.08195	H23C	0.24819	109.339
O2	C3	0.13587	C2	0.23391	31.864
O2	C3	0.13587	C4	0.24221	28.179
O2	C3	0.13587	H23C	0.24819	92.121
O2	C2	0.23391	C4	0.24221	60.033
O2	C2	0.23391	H23C	0.24819	81.083
O2	C4	0.24221	H23C	0.24819	100.564
O3	H3A	0.08905	C4	0.13819	106.205
O3	H3A	0.08905	C3	0.23628	135.018
O3	H3A	0.08905	H2A	0.23641	161.689

O3	H3A	0.08905	H4A	0.23736	68.895
O3	H3A	0.08905	C5	0.24122	79.16
O3	C4	0.13819	C3	0.23628	31.275
O3	C4	0.13819	H2A	0.23641	76.167
O3	C4	0.13819	H4A	0.23736	119.491
O3	C4	0.13819	C5	0.24122	29.1
O3	C3	0.23628	H2A	0.23641	44.899
O3	C3	0.23628	H4A	0.23736	133.007
O3	C3	0.23628	C5	0.24122	60.36
O3	H2A	0.23641	H4A	0.23736	126.403
O3	H2A	0.23641	C5	0.24122	105.213
O3	H4A	0.23736	C5	0.24122	99.824
O4	H4A	0.08616	C8	0.13738	99.652
O4	H4A	0.08616	H5A	0.22243	159.701
O4	H4A	0.08616	C7	0.23715	128.359
O4	H4A	0.08616	C9	0.23858	67.386
O4	C8	0.13738	H5A	0.22243	97.931
O4	C8	0.13738	C7	0.23715	29.094
O4	C8	0.13738	C9	0.23858	32.469
O4	H5A	0.22243	C7	0.23715	70.391
O4	H5A	0.22243	C9	0.23858	128.76
O4	C7	0.23715	C9	0.23858	61.552
C5	H5A	0.09294	C4	0.13796	119.926

C5	H5A	0.09294	C6	0.14023	119.991
C5	H5A	0.09294	C3	0.24009	149.496
C5	H5A	0.09294	H3A	0.24091	71.197
C5	H5A	0.09294	O3	0.24122	90.776
C5	H5A	0.09294	C1	0.2416	150.287
C5	H5A	0.09294	C7	0.24942	89.336
C5	C4	0.13796	C6	0.14023	120.083
C5	C4	0.13796	C3	0.24009	29.662
C5	C4	0.13796	H3A	0.24091	49.49
C5	C4	0.13796	O3	0.24122	29.153
C5	C4	0.13796	C1	0.2416	89.786
C5	C4	0.13796	C7	0.24942	150.737
C5	C6	0.14023	C3	0.24009	90.468
C5	C6	0.14023	H3A	0.24091	166.146
C5	C6	0.14023	O3	0.24122	149.229
C5	C6	0.14023	C1	0.2416	30.298
C5	C6	0.14023	C7	0.24942	30.654
C5	C3	0.24009	H3A	0.24091	78.956
C5	C3	0.24009	O3	0.24122	58.799
C5	C3	0.24009	C1	0.2416	60.179
C5	C3	0.24009	C7	0.24942	121.107
C5	H3A	0.24091	O3	0.24122	21.286
C5	H3A	0.24091	C1	0.2416	138.063

C5	H3A	0.24091	C7	0.24942	158.624
C5	O3	0.24122	C1	0.2416	118.936
C5	O3	0.24122	C7	0.24942	179.499
C5	C1	0.2416	C7	0.24942	60.952
O6	H6A	0.08197	C11	0.13545	109.439
O6	H6A	0.08197	C12	0.23596	138.982
O6	H6A	0.08197	C10	0.23894	77.589
O6	C11	0.13545	C12	0.23596	29.546
O6	C11	0.13545	C10	0.23894	31.853
O6	C12	0.23596	C10	0.23894	61.399
O7	H7A	0.0819	C13	0.13592	109.524
O7	H7A	0.0819	C12	0.23397	138.751
O7	H7A	0.0819	C14	0.24017	81.667
O7	C13	0.13592	C12	0.23397	32.148
O7	C13	0.13592	C14	0.24017	29.767
O7	C12	0.23397	C14	0.24017	61.914
C12	H12A	0.09301	C11	0.1357	120.275
C12	H12A	0.09301	C13	0.13916	120.47
C12	H12A	0.09301	O7	0.23397	89.158
C12	H12A	0.09301	O6	0.23596	90.79
C12	H12A	0.09301	C10	0.24247	150.693
C12	H12A	0.09301	C14	0.24395	149.449
C12	C11	0.1357	C13	0.13916	119.255

C12	C11	0.1357	O7	0.23397	150.567
C12	C11	0.1357	O6	0.23596	29.485
C12	C11	0.1357	C10	0.24247	30.419
C12	C11	0.1357	C14	0.24395	90.276
C12	C13	0.13916	O7	0.23397	31.313
C12	C13	0.13916	O6	0.23596	148.739
C12	C13	0.13916	C10	0.24247	88.836
C12	C13	0.13916	C14	0.24395	28.98
C12	O7	0.23397	O6	0.23596	179.946
C12	O7	0.23397	C10	0.24247	120.148
C12	O7	0.23397	C14	0.24395	60.291
C12	O6	0.23596	C10	0.24247	59.905
C12	O6	0.23596	C14	0.24395	119.761
C12	C10	0.24247	C14	0.24395	59.857
N1	C16	0.13375	C17	0.13851	105.803
N1	C16	0.13375	C21	0.14644	126.989
N1	C16	0.13375	H21B	0.20006	132.881
N1	C16	0.13375	H21A	0.2001	100.203
N1	C16	0.13375	H21C	0.20017	138.674
N1	C16	0.13375	H16A	0.2003	22.89
N1	C16	0.13375	C20	0.21902	68.725
N1	C16	0.13375	N4	0.22477	33.212
N1	C17	0.13851	C21	0.14644	126.952

N1	C17	0.13851	H21B	0.20006	110.816
N1	C17	0.13851	H21A	0.2001	153.875
N1	C17	0.13851	H21C	0.20017	110.9
N1	C17	0.13851	H16A	0.2003	128.693
N1	C17	0.13851	C20	0.21902	37.078
N1	C17	0.13851	N4	0.22477	72.592
N1	C21	0.14644	H21B	0.20006	26.835
N1	C21	0.14644	H21A	0.2001	26.923
N1	C21	0.14644	H21C	0.20017	26.901
N1	C21	0.14644	H16A	0.2003	104.179
N1	C21	0.14644	C20	0.21902	163.551
N1	C21	0.14644	N4	0.22477	159.882
N1	H21B	0.20006	H21A	0.2001	46.105
N1	H21B	0.20006	H21C	0.20017	46.117
N1	H21B	0.20006	H16A	0.2003	114.067
N1	H21B	0.20006	C20	0.21902	140.346
N1	H21B	0.20006	N4	0.22477	151.392
N1	H21A	0.2001	H21C	0.20017	46.117
N1	H21A	0.2001	H16A	0.2003	77.331
N1	H21A	0.2001	C20	0.21902	168.651
N1	H21A	0.2001	N4	0.22477	133.379
N1	H21C	0.20017	H16A	0.2003	117.729
N1	H21C	0.20017	C20	0.21902	144.73

N1	H21C	0.20017	N4	0.22477	161.307
N1	H16A	0.2003	C20	0.21902	91.615
N1	H16A	0.2003	N4	0.22477	56.102
N1	C20	0.21902	N4	0.22477	35.514
N2	C19	0.13803	C18	0.14076	126.989
N2	C19	0.13803	C22	0.14848	116.293
N2	C19	0.13803	H22B	0.20194	127.243
N2	C19	0.13803	H22C	0.202	126.109
N2	C19	0.13803	H22A	0.20206	89.686
N2	C19	0.13803	O10	0.22816	27.399
N2	C19	0.13803	O9	0.23077	153.694
N2	C19	0.13803	C17	0.23311	92.124
N2	C19	0.13803	N3	0.2352	30.845
N2	C18	0.14076	C22	0.14848	116.693
N2	C18	0.14076	H22B	0.20194	101.727
N2	C18	0.14076	H22C	0.202	101.436
N2	C18	0.14076	H22A	0.20206	143.304
N2	C18	0.14076	O10	0.22816	154.386
N2	C18	0.14076	O9	0.23077	26.739
N2	C18	0.14076	C17	0.23311	34.875
N2	C18	0.14076	N3	0.2352	96.144
N2	C22	0.14848	H22B	0.20194	26.637
N2	C22	0.14848	H22C	0.202	26.666

N2	C22	0.14848	H22A	0.20206	26.613
N2	C22	0.14848	O10	0.22816	88.914
N2	C22	0.14848	O9	0.23077	90.012
N2	C22	0.14848	C17	0.23311	151.452
N2	C22	0.14848	N3	0.2352	147.114
N2	H22B	0.20194	H22C	0.202	45.691
N2	H22B	0.20194	H22A	0.20206	45.714
N2	H22B	0.20194	O10	0.22816	102.075
N2	H22B	0.20194	O9	0.23077	76.462
N2	H22B	0.20194	C17	0.23311	133.874
N2	H22B	0.20194	N3	0.2352	152.061
N2	H22C	0.202	H22A	0.20206	45.685
N2	H22C	0.202	O10	0.22816	101.898
N2	H22C	0.202	O9	0.23077	77.603
N2	H22C	0.202	C17	0.23311	131.533
N2	H22C	0.202	N3	0.2352	149.456
N2	H22A	0.20206	O10	0.22816	62.301
N2	H22A	0.20206	O9	0.23077	116.617
N2	H22A	0.20206	C17	0.23311	177.089
N2	H22A	0.20206	N3	0.2352	120.522
N2	O10	0.22816	O9	0.23077	178.383
N2	O10	0.22816	C17	0.23311	119.522
N2	O10	0.22816	N3	0.2352	58.243

N2	O9	0.23077	C17	0.23311	61.613
N2	O9	0.23077	N3	0.2352	122.859
N2	C17	0.23311	N3	0.2352	61.284
N4	C16	0.13456	C20	0.13546	102.91
N4	C16	0.13456	H16A	0.20106	22.799
N4	C16	0.13456	H2A	0.20389	119.534
N4	C16	0.13456	N1	0.22477	32.984
N4	C16	0.13456	C17	0.22601	68.771
N4	C16	0.13456	N3	0.24389	129.682
N4	C20	0.13546	H16A	0.20106	125.708
N4	C20	0.13546	H2A	0.20389	137.444
N4	C20	0.13546	N1	0.22477	69.926
N4	C20	0.13546	C17	0.22601	34.14
N4	C20	0.13546	N3	0.24389	26.777
N4	H16A	0.20106	H2A	0.20389	96.759
N4	H16A	0.20106	N1	0.22477	55.783
N4	H16A	0.20106	C17	0.22601	91.57
N4	H16A	0.20106	N3	0.24389	152.476
N4	H2A	0.20389	N1	0.22477	152.449
N4	H2A	0.20389	C17	0.22601	171.29
N4	H2A	0.20389	N3	0.24389	110.696
N4	N1	0.22477	C17	0.22601	35.787
N4	N1	0.22477	N3	0.24389	96.702

N4	C17	0.22601	N3	0.24389	60.917
C16	H16A	0.093	N1	0.13375	123.097
C16	H16A	0.093	N4	0.13456	123.098
C16	H16A	0.093	C20	0.21119	161.793
C16	H16A	0.093	C17	0.21717	160.953
C16	N1	0.13375	N4	0.13456	113.804
C16	N1	0.13375	C20	0.21119	75.108
C16	N1	0.13375	C17	0.21717	37.856
C16	N4	0.13456	C20	0.21119	38.697
C16	N4	0.13456	C17	0.21717	75.949
C16	C20	0.21119	C17	0.21717	37.253
C21	H21B	0.09584	H21C	0.09608	109.53
C21	H21B	0.09584	H21A	0.09608	109.46
C21	H21B	0.09584	N1	0.14644	109.555
C21	H21C	0.09608	H21A	0.09608	109.345
C21	H21C	0.09608	N1	0.14644	109.5
C21	H21A	0.09608	N1	0.14644	109.439
C22	H22B	0.09604	H22A	0.09606	109.55
C22	H22B	0.09604	H22C	0.09615	109.377
C22	H22B	0.09604	N2	0.14848	109.482
C22	H22B	0.09604	C19	0.24342	122.459
C22	H22B	0.09604	C18	0.24624	92.775
C22	H22A	0.09606	H22C	0.09615	109.382

C22	H22A	0.09606	N2	0.14848	109.569
C22	H22A	0.09606	C19	0.24342	79.02
C22	H22A	0.09606	C18	0.24624	140.279
C22	H22C	0.09615	N2	0.14848	109.466
C22	H22C	0.09615	C19	0.24342	121.161
C22	H22C	0.09615	C18	0.24624	92.437
C22	N2	0.14848	C19	0.24342	30.555
C22	N2	0.14848	C18	0.24624	30.711
C22	C19	0.24342	C18	0.24624	61.259
C23	H23C	0.09596	H23A	0.09596	109.52
C23	H23C	0.09596	H23B	0.09599	109.572
C23	H23C	0.09596	N3	0.14754	109.473
C23	H23C	0.09596	C19	0.24584	122.581
C23	H23C	0.09596	C20	0.24731	93.95
C23	H23A	0.09596	H23B	0.09599	109.456
C23	H23A	0.09596	N3	0.14754	109.432
C23	H23A	0.09596	C19	0.24584	80.68
C23	H23A	0.09596	C20	0.24731	138.006
C23	H23B	0.09599	N3	0.14754	109.374
C23	H23B	0.09599	C19	0.24584	119.835
C23	H23B	0.09599	C20	0.24731	93.702
C23	N3	0.14754	C19	0.24584	28.784
C23	N3	0.14754	C20	0.24731	28.574

C23	C19	0.24584	C20	0.24731	57.335
